# Impact of Long‐Term Fasting on Skeletal Muscle: Structure, Energy Metabolism and Function Using ^31^P/^1^H MRS and MRI

**DOI:** 10.1002/jcsm.13773

**Published:** 2025-04-11

**Authors:** Antoine Naëgel, Magalie Viallon, Hélène Ratiney, Thu Nguyen, Benjamin Leporq, Djahid Kennouche, Thomas Grenier, Franziska Grundler, Robin Mesnage, Jean‐Michel Guy, Robin Schultze, Françoise Wilhelmi de Toledo, Pierre Croisille

**Affiliations:** ^1^ Univ Lyon, UJM‐Saint‐Etienne, INSA, CNRS UMR 5520, INSERM U1206, CREATIS Saint‐Etienne France; ^2^ Siemens Healthcare SAS Saint‐Denis France; ^3^ Department of Radiology University Hospital Saint‐Etienne Saint‐Etienne France; ^4^ Laboratoire Interuniversitaire de Biologie de la Motricité Université Jean Monnet Saint‐Etienne, Lyon 1, Université Savoie Mont‐Blanc Saint‐Etienne France; ^5^ Buchinger Wilhelmi Clinic Überlingen Germany; ^6^ Department of Nutritional Sciences, School of Life Course Sciences, Faculty of Life Sciences and Medicine King's College London London UK; ^7^ Le Clos Champirol Reeducation Saint‐Priest‐en‐Jarez France; ^8^ Mettnau, Werner‐Messmer‐Klinik Radolfzell am Bodensee Germany

**Keywords:** fasting, magnetic resonance imaging (MRI), magnetic resonance spectroscopy (MRS), muscle metabolism, muscle strength, substrate utilization

## Abstract

**Background:**

Fasting shows promise for public health, but concerns about muscle loss hinder its acceptance, particularly among the elderly. We explored the impact of long‐term fasting (12 days, 250 kcal/day) on muscle structure, metabolism and performance.

**Methods:**

We prospectively assessed muscle volume, composition, relaxometry data and lipid metabolism in 32 subjects (16 men; 50% over 50 years old) before fasting, at the end of fasting and 1 month post‐fasting. Techniques included high‐resolution 3D Dixon MR imaging, multiecho CSE and single‐voxel MR spectroscopy. Dynamic ^31^P‐MRS, quantitative MRI, maximal voluntary contraction (MVC) measurements and exercise testing (VO_2_peak) were repeated throughout the protocol.

**Results:**

Although the average body weight loss was 5.9 kg (7.4%, *p* < 0.001), the skeletal muscle volume change measured on the right calf muscle was 271 mL (5.4%, *p* < 0.001). This closely aligns with expected losses of glycogen (1%–2%) and bound water (3%–4%), estimated to total 404–505 mL. MVC (anaerobic lactic metabolism) remained preserved in both thighs and calf muscles, regardless of sex or age. Unchanged T2 showed that fasting did not induce structural or inflammatory changes. MRI/MRS revealed fat redistribution among tissues, with subcutaneous fat decrease (by 417.2 cm^3^, *p* < 0.01) and total fat fraction increase (by 0.2%, *p* < 0.05) in muscle. The intramyocellular lipid pool increased by 2.2 times (*p* < 0.05), whereas the extracellular lipid pool decreased to 1.4 times (*p* < 0.05), revealing rapid lipid trafficking and adaptation. During fasting, the T2* value increased by 1.2 ms (*p* < 0.001), likely because of changes in the configuration of intracellular lipid droplets, with an increased proportion of lipid droplets of smaller size, optimizing accessibility of lipid fuels and mitochondrial FA. Exercise testing (VO_2_peak) showed no change in maximal oxygen uptake, but fat oxidation improved with a 10% decrease in the exercise respiratory exchange ratio (*p* < 0.001). Mitochondrial oxidative capacity and PCr resynthesis rates in muscle were maintained. Females improved their mitochondrial function by D + 12, with τPCr decreasing to 29.61 s (*p* < 0.01), surpassing males and demonstrating better fat oxidation capabilities.

**Conclusions:**

Long‐term fasting did not alter muscle metabolism or performance, nor induced structural or inflammatory changes. The decrease in muscle volume is minor when accounting for glycogen and water depletion during fasting. Fat is relocated to the intracellular compartment of myocytes. Both anaerobic and aerobic metabolic pathways remain unchanged after 12 days of fasting in both sexes and older subjects. This suggests that human muscles, like those in animals, have evolved to withstand seasonal food shortages and endure long fasting periods.

## 1 Introduction

Human life expectancy has steadily increased in the past centuries thanks to improved medical care. However, the modern unhealthy Western lifestyle has hindered this progress by promoting an epidemic of chronic metabolic and inflammatory diseases [1]. This has led to research into therapeutic strategies that promote healthy ageing, with fasting (intermittent and long‐term) and calorie restriction diets emerging as the most promising approaches to extending healthy lifespan [2, 3]. Fasting has always been practiced in free‐living animals to cope with seasonal changes in food availability and in humans for religious and spiritual reasons. However, the development of fasting as a therapeutic approach has led to a debate on possible muscle loss [4, 5]. This is because the body can mobilize proteins to supply amino acids to the liver and kidney for neoglucogenesis, ensuring that the brain and other organs receive the glucose they need to function normally.

Concerns about loss of skeletal muscle during fasting mainly originate from studies conducted in the 1960s on the energy metabolism of obese individuals undergoing long periods of water‐only fasting for weight loss [6, 7]. Whether fasting affects muscle amino acid metabolism remains controversial as some studies report that net muscle amino acid release increases with fasting [8], whereas others report this is unaltered. In addition, although studies have shown that the body metabolizes amino acids during fasting, it is unclear whether these amino acids come from the muscles and other organs that are profoundly remodelled by autophagy [9]. Furthermore, studies showing that protein use can be different between lean subjects and obese subjects and, according to the duration of the fasting period, prompt the need for new studies in healthy subjects from both sexes and different age groups [5].

The density of proteins in the muscular tissue has long led to the assumption that the protein utilization for neoglucogenesis, which is maximal in the first days of fasting, was provided by the mobilization of proteins from skeletal muscles. Natural cycling of muscle mass occurs between eating and fasting in wild animals, such as king penguins [10]. In this case, protein usage during long periods of fasting remains limited because of the activation of protein‐sparing mechanisms when ketone bodies are generated from fatty acids [11]. Muscle mass loss is recovered when the animal is refed, as reflected by decreased myostatin levels in fasting animals. The same is observed in humans after the food reintroduction [4]. The reversibility of these modifications suggests that any changes in muscle metabolism in fasting humans could only be transitory adaptations to the fasting metabolism. We hypothesize that amino acids utilized to produce glucose during fasting can come from other tissues through autophagy. This is clear in laboratory rodents, for which it is shown that liver‐specific autophagy provided amino acids for neoglucogenesis to maintain blood glucose levels [12]. Although lean mass changes have been reported using indirect estimates after prolonged fasting [4, 13, 14], no study has directly quantified muscle composition changes in fasting humans. It remains unclear whether these changes are permanent damage or reversible effects [15].

The safety of natural variations in muscle mass in animals during seasonal changes in food availability further suggests that we need to consider factors beyond just composition to fully understand how muscle function may be affected. The impacts of fasting on muscle metabolism, performance and integrity—beyond just changes in muscle composition—are still largely unexplored. Only a recent prospective trial in healthy men confirmed a protein loss occurring in early fast, decreasing as ketogenesis increases, but concluded that fasting combined with physical activity does not seem to impact muscle function negatively [4]. Thus, new studies using advanced non‐invasive technologies are needed to investigate the impact of fasting on the human body with a particular focus on skeletal muscle and improve our understanding of both possible functional and compositional changes. Dynamic ^31^P‐magnetic resonance spectroscopy (^31^P‐MRS) and proton MR imaging (^1^H‐MRI) can offer unrivalled non‐invasive longitudinal quantification capabilities of organ changes from macroscopic anatomy to microscopic metabolic changes and function in muscle, as performed in the CALERIE 2 study of the effects of caloric restriction on mitochondrial function [16, 17].

In this prospective longitudinal study, we use quantitative MRI and MRS to investigate macroscopic to metabolic muscle coupled changes in a representative population of healthy men and women before, at the end of a 12‐day fast and physical activity programme and 1 month after food reintroduction.

## 2 Materials and Methods

### 2.1 Participants

This prospective, monocentric, single‐arm interventional study enrolled 16 men and 16 women (44 ± 14 years; 26.2 ± 0.9 kg/m^2^) who fasted for 12 days and maintained up to 3 h of daily low‐intensity physical activity. The fasting protocol and the sample size calculation are detailed in the GENESIS (lonG tErm FastiNg multi‐systEm adaptationS In humanS) study protocol [18]. All measurements, including muscular strength measurements, activity monitoring, cardiopulmonary capacity, muscular volume, MR relaxometry, ^1^H and ^31^P MR spectroscopy and fat composition, were performed at three time points: before (D − 1), at the end (D + 12) and 1 month (M + 1) after the end of fasting. The Baden‐Württemberg Medical Council approved the study (F‐2021‐075, 26 July 2021) and the study is registered at ClinicalTrials.gov (NCT05031598). A signed informed written consent was obtained from all participants.

### 2.2 Muscular Strength and Volume Measurements

The maximal voluntary contraction (MVC) of the quadriceps and calf muscles, respectively obtained by knee extension and plantar flexion, were measured on the same day of each MR session (D − 1, D + 12 and M + 1) (see Supporting Information for details). Thigh and calf muscle volume were obtained using an automatic segmentation process on T1 Dixon Water‐only (T1W) and Fat‐only (T1F) 3D MR images using a multiatlas segmentation with joint label fusion [19, 20] in all 32 subjects at the three observation time points. T2 in the calf muscle was further extracted from the T2 maps as described by Nguyen et al. [19].

### 2.3 Activity Monitoring and Cardiopulmonary Exercise Testing

Activity monitoring was quantified continuously, starting 2 weeks before fasting until 2 weeks after refeeding. A maximal cardiopulmonary exercise cycle ergometer test was also conducted at each MR session (see Supporting Information for details).

### 2.4 MR Protocol

The study used a Siemens MAGNETOM 3T Prisma MRI (Erlangen, Germany) to perform High‐resolution anatomical 3D isotropic Dixon sequence, quantitative T2 mapping and ^1^H‐MRS acquisitions on the thigh and dynamic ^31^P‐MRS acquisitions on the calf muscle.

### 2.5 MR Relaxometry, Tissue Fat Composition and ^1^H‐MRS

All the measures detailed below were performed on the thigh. Table S1 summarizes the main parameters of the MR sequences that constitute the MR protocol ran at each time point of the interventional study. A 3D gradient multiecho chemical‐shift‐encoded imaging (CSE‐MRI) sequence was used to extract fat fraction map and relaxometry data [21]. The determination of subcutaneous adipose tissue (SAT) and bone marrow adipose tissue (BMAT) is detailed in the following article [22]. Briefly, the parameters PDFF (proton density fat fraction) and T2* can be determined using mathematical modelling of the signals obtained from a CSE‐MRI. The post‐processing algorithm is divided into two steps: The first consists of correcting the phase and heterogeneity of the B0 field, and the second concerns the quantification of fatty acid composition. For this second step, it is essential to know the structure of the fatty acids, expressed in terms of the number of double bonds (ndb), double bonds interrupted by a methylene group (nmidb) and chain length (CL) [23]. From this information, saturated fatty acid (SFA), monounsaturated fatty acid (MUFA) and polyunsaturated fatty acid (PUFA) fractions can be derived.

We derived lipid metabolism from single‐voxel STEAM ^1^H‐MRS acquisitions from a 30 × 30 × 30 mm^3^ voxel located in the vastus medialis head of the quadriceps muscle (see Figure S1B). ^1^H‐MRS metabolites were quantified after fitting the acquired spectra using the standard LCModel [24], to extract intra‐myocellular lipids (IMCL)_CH2_ resonances (IMCL at 1.3 ppm), extra‐myocellular lipids (EMCL)_CH2_ (EMCL at 1.5 ppm) and taurine (Tau) at 3.4 ppm. We also used choline (Cho) to refer to any trimethylammonium (TMA) compounds as proposed by others [25]. We calculated relative concentrations for all metabolites using the creatine resonance (Cr) at 3.0 ppm as a reference [26].

### 2.6 Dynamic ^31^P‐MRS

**FIGURE 1:**
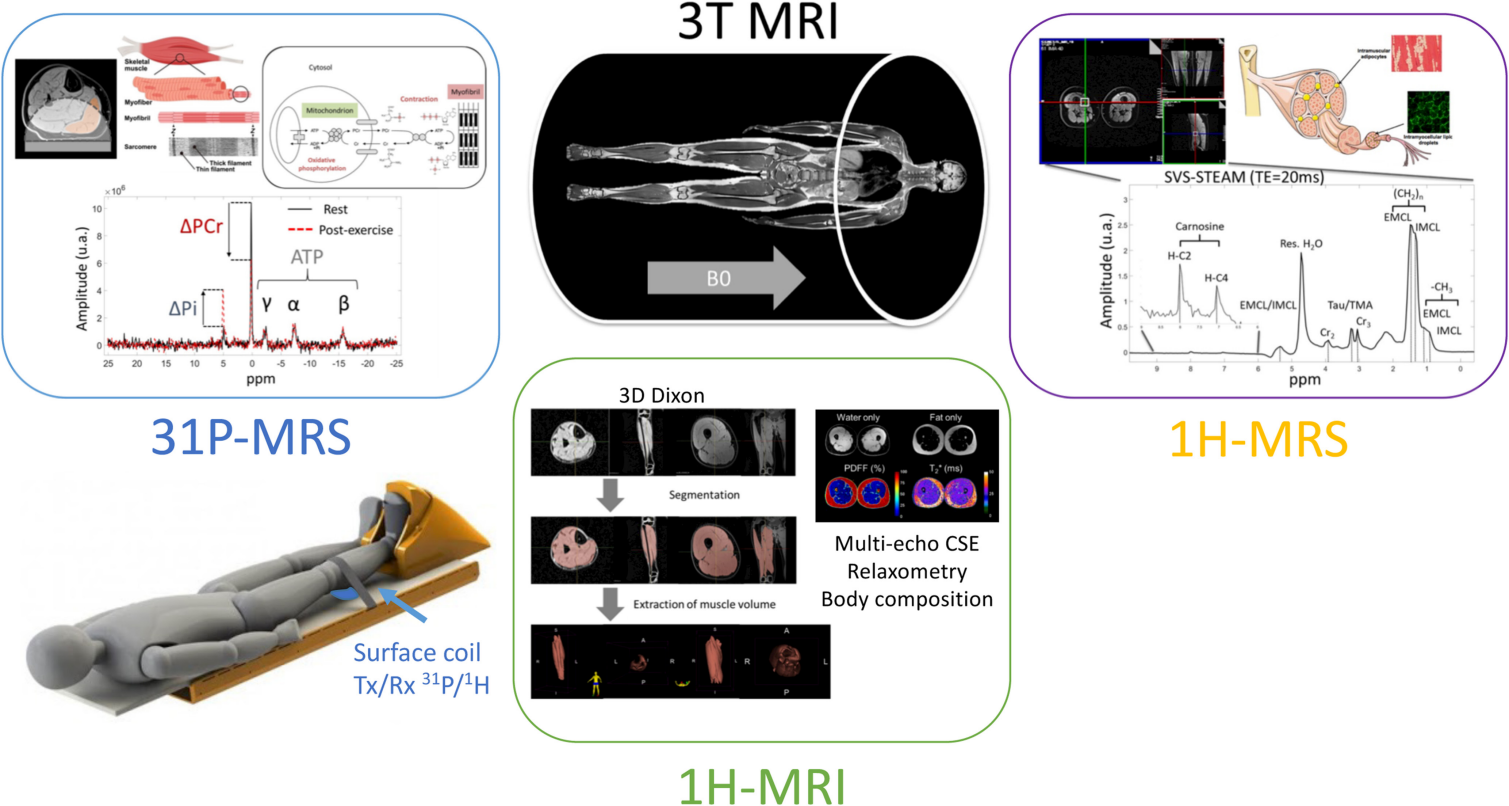
Global overview of the MRI and MRS protocol including: (i) ^31^P dynamic MRS coupled to exercise using an MR ergometer, (ii) ^1^H‐MRS using SVS‐STEAM MRS to investigate lipid composition and IMCL/EMCL sub‐composition, (iii) ^1^H high‐resolution 3D MRI for muscle volume quantification and CSE‐MRI for advanced tissue characterization.

**FIGURE 2:**
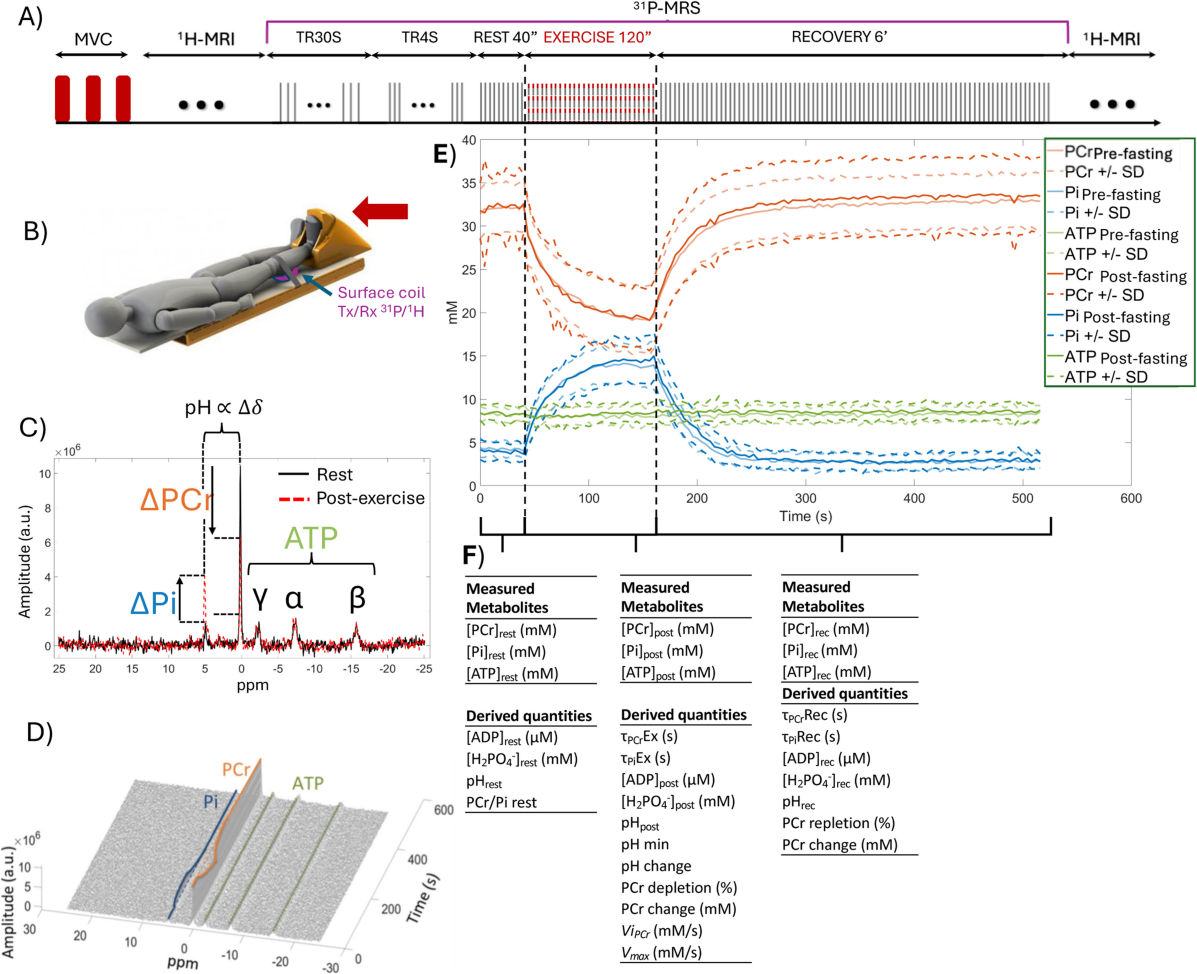
Illustration displaying (A) the different steps and time course of the calf muscle exercise using the ergometer within the MR scanner (B); (C) typical ^31^P spectrum with highlighted main peaks of metabolites of interest, (D) 3D overview of SRM‐^31^P dynamic time acquisition; (E) derived temporal curves of metabolites over time. (F) Metabolites or quantities derived from modelling are listed below the graph from each protocol phase.

We explored energy metabolism by dynamics FID ^31^P‐MRS acquisitions (Table S1) on volunteers' calf muscles with saturation bands to minimize the signal from unstressed muscles outside the volume of interest and bone (Figure S1D). The platform featured an MRI‐compatible ergometer (ErgoSpect GmbH, Innsbruck, Austria) with a calf module. Subjects were lying supine with the dual ^1^H/^31^P surface coil (RAPID Biomedical GmbH, Rimpar, Germany) under their calf muscle (Figures 1 and 2).

We calculated ^31^P‐MRS metabolites concentrations and associated time constants in agreement with the latest recommendations [27, 28, 29]. Quantification was performed using the QUEST (QUantitation based on QUantum ESTimation) method [30]. The millimolar concentration of phosphorus metabolites was evaluated based on the standard assumption that [ATP] is 8.2 mmol/L of cell water [31], as recommended by the latest guidelines [27]. The concentrations of phosphocreatine (PCr), inorganic phosphate (Pi), adenosine triphosphate (ATP) and phosphodiesters (PDE) metabolites were extracted at rest, post‐exercise and after recovery. The concentration of ADP and diprotonated phosphate [H_2_PO_4_^−^] was derived according to [27, 32]. The intracellular pH is calculated using the Henderson–Hasselbalch equation, based on the difference in chemical shift δ between Pi and PCr found in the ^31^P‐MRS spectrum [27]. We also calculated the PCr depletion (percentage) during plantar exercise, the time constants of PCr (τPCrEx and τPCrRec) and Pi (τPiEx and τPiRec) during exercise, during recovery, as well as the initial recovery rate of PCr (ViPCr) and the maximum aerobic capacity (Vmax) according to [27, 33]. During recovery, τPCrRec reflects the rate of PCr resynthesis and mitochondrial oxidative capacity to regenerate ATP, with lower values meaning faster recovery kinetics and higher oxidative capacity. ViPCr measures PCr recovery kinetics; faster PCr recovery kinetics means improved maximal mitochondrial ATP production. The maximum aerobic capacity (Vmax) represents the theoretical maximum velocity at which muscle can synthesize ATP via oxidative phosphorylation and is dependent on the number and function of mitochondria in muscles.

### 2.7 Statistics

All variables were screened for normality using the Shapiro–Wilk test and reported as the mean (SD) or mean [95% CI] when appropriate. To examine the effects of age and sex (between‐subjects factors) and time (within‐subjects factor) and their interactions, we used a three‐way repeated‐measures mixed‐effects model approach with a compound symmetry covariance matrix and restricted maximum likelihood (REML) fitting. A Geisser–Greenhouse correction was applied to account for the sphericity assumption violation. When applicable, pairwise post‐hoc comparisons were obtained with a Tukey test to correct for multiple comparisons. For all analyses, significance was accepted at *p* < 0.05. The statistical analyses were conducted using Prism 10.1 (La Jolla, CA, USA) and Stata 18 (College Station, TX).

## 3 Results

### 3.1 Demographic Data and Total Body Weight Changes

Baseline anthropometric data on the subject's characteristics are given in Table S2. As expected, fasting led to a significant weight loss (*p* = 0.002) without a significant effect of sex or age (*p* = 0.120 and *p* = 0.934, respectively) and no two‐way interactions. The average total body weight loss from baseline was −5.9 [−6.4; −5.5] kg at D + 12 and −4.1 [−4.8; −3.3] kg at M + 1 (Table 1).

**TABLE 1:** Weight, thigh and calf skeletal muscle volumes, thigh and calf maximum voluntary contraction (MVC) force measurements (mean (SD), on overall, and stratified by sex and age). The table presents the effects of time, sex and age using a repeated‐measures mixed‐effects model. When applicable, pairwise post‐hoc comparisons used a Tukey test to correct for multiple comparisons. For all analyses, significance was accepted at *p* < 0.05. Legend for post‐hoc analysis: D + 12 and M + 1 versus D − 1: * = *p* < 0.05; ** = *p* < 0.01; *** = *p* < 0.001.

Variables	Time				Sex					Age					Interactions		
															Time * sex	Time * age	Sex * age
	D − 1	D + 12	M + 1	*p*		D − 1	D + 12	M + 1	*p*		D − 1	D + 12	M + 1	*p*	*p*	*p*	*p*
Weight (kg)	78.8 (13.1)	72.9 (12.2)***	74.7 (12.2)***	0.002	Male	85.2 (12.1)	78.4 (11.9)	80.6 (11.3)	0.12	Young	80.0 (11.1)	74.0 (10.5)	74.5 (9.9)	0.934	0.051	0.072	0.729
					Female	72.8 (9.7)	67.7 (9.1)	68.8 (8.8)		Old	77.7 (15.2)	71.9 (14.1)	74.9 (14.6)				
Thigh muscle volume (mL)	3617 (965)	3403 (890)***	3492 (953)***	< 0.001	Male	4444 (726)	4175 (639)***	4299 (712)***	< 0.001	Young	3786 (997)	3556 (894)	3691 (963)	0.068	0.051	0.078	0.618
					Female	2886 (367)	2722 (355)***	2797 (383)**		Old	3424 (922)	3228 (883)	3265 (921)				
Thigh MVC (N * m)	224.2 (76.6)	231.1 (81.0)	226.7 (80.0)	0.138	Male	263.2 (57.4)	273.7 (66.8)	267.1 (65.4)	< 0.001	Young	249.4 (72.1)	254.9 (74.8)	245.4 (75.0)	0.011	0.53	0.078	0.198
					Female	187.5 (75.6)	191.0 (73.7)	186.3 (74.0)		Old	197.4 (74.2)	205.8 (81.9)	207.9 (82.8)				
Calf muscle volume (mL)	1433 (285)	1376 (276)***	1390 (279)***	< 0.001	Male	1643 (254)	1567 (266)***	1600 (247)***	< 0.001	Young	1472 (295)	1416 (278)	1440 (285)	0.229	0.007	0.166	0.42
					Female	1247 (150)	1208 (145)***	1205 (140)***		Old	1389 (276)	1330 (275)	1335 (270)				
Calf MVC (N * m)	680.1 (122.9)	712.4 (84.9)	706.8 (71.1)	0.089	Male	725.7 (78.9)	737.1 (60.1)	739.5 (37.9)	0.016	Young	699.0 (92.5)	723.7 (83.2)	707.2 (64.8)	0.403	0.405	0.394	0.42
					Female	637.1 (142.5)	689.1 (99.1)	676.0 (81.7)		Old	660.0 (149.1)	700.3 (87.7)	706.3 (79.4)				

### 3.2 Muscular Volumes and Strength Measurements

**FIGURE 3:**
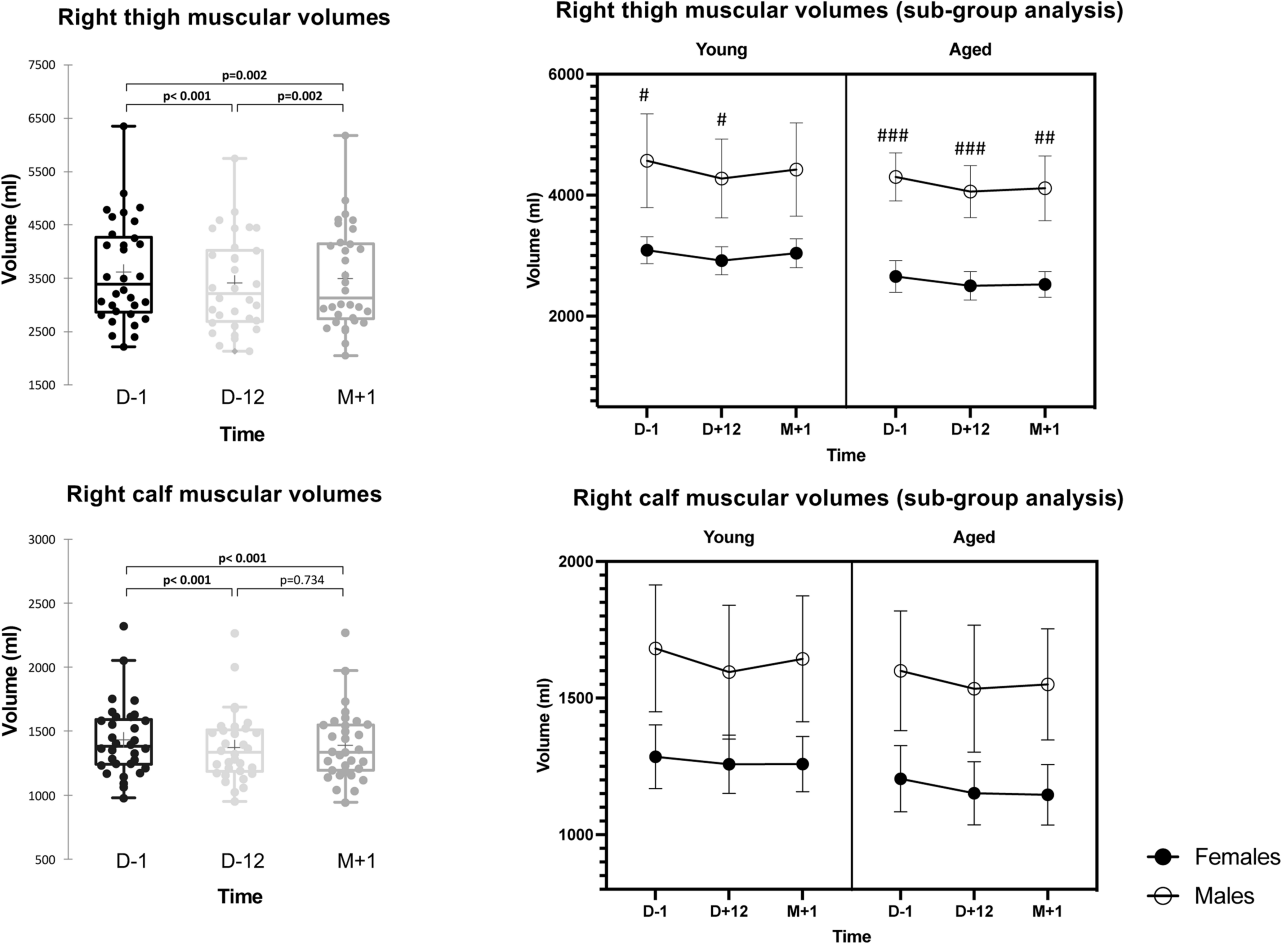
Thigh and calf muscular volume measurements obtained before (D − 1), at the end (D + 12) and 1 month (M + 1) after the end of fasting; left: global results, right: age and sex subgroup results (graph data are mean ± 95% CI; post‐hoc comparisons between males and females; # = *p* < 0.05; ## = *p* < 0.01; ### = *p* < 0.001).

Results are detailed in Table 1. We observed a significant effect of time (fasting) on right thigh muscular volumes (*p* < 0.001), as well as an effect of sex (*p* < 0.001) but no influence of age (*p* = 0.068) (see also Figure 3). We observed a similar pattern on right calf muscular volumes but with a time * sex interaction (*p* = 0.007) and no influence of age on calf muscle volumes (*p* = 0.229). At D + 12, thigh muscle volume loss was −269[−355, −184] mL in male (*p* < 0.001) and −164[−211, −119] mL (*p* < 0.001) in female. Between D + 12 and M + 1, the muscle volume gain was significant in females +75.6[9, 142] mL (*p* = 0.02) but not in males (*p* = 0.08). Calf muscle volume loss at D + 12 was −76[−103, −50] mL (*p* < 0.001) in male and −39[−61, −17] mL (*p* < 0.001) in female. Between D + 12 and M + 1, males gained +33[2, 64] mL (*p* = 0.035), whereas females remained unchanged with −2.3[−13.7, 9.1] mL. After refeeding at M + 1, muscle volume difference for the right calf muscle remained overall at 168 mL (3.3%), 208 mL (3.4%) in males and 131 mL (3.2%) in females (Table 2).

**TABLE 2:** Absolute (mL) and relative (%) skeletal muscle volume differences (right thigh, right calf, right lower limb).

	Group	Volume D − 1 (mL)	Volume D + 12 (mL)	Volume M + 1 (mL)	Absolute volume difference D + 12 vs. D − 1 (mL)	Absolute volume difference M + 1 vs. D − 1 (mL)	Relative volume difference D + 12 vs. D − 1 (%)	Relative volume difference M + 1 vs. D − 1 (%)
Right thigh	All	3617	3403	3492	−214	−125	−5.9	−3.5
	Male	4444	4175	4279	−269	−165	−6.1	−3.7
	Female	2886	2722	2797	−164	−89	−5.7	−3.1
Right calf	All	1433	1376	1390	−57	−43	−4.0	−3.0
	Male	1643	1567	1600	−76	−43	−4.6	−2.6
	Female	1247	1208	1205	−39	−42	−3.1	−3.4
Right lower limb	All	5050	4779	4882	−271	−168	−5.4	−3.3
	Male	6087	5742	5879	−345	−208	−5.7	−3.4
	Female	4133	3930	4002	−203	−131	−4.9	−3.2

Muscle strength, evaluated by MVC measures, remained stable over time during fasting for quadriceps and calf muscles (*p* = 0.138 and *p* = 0.089, respectively) (Table 1). We observed significant differences between males and females (quadriceps: *p* < 0.001 and calf: *p* = 0.016). In contrast, a difference between the young and aged groups existed only for thigh measures (*p* = 0.011), without any two‐way interactions.

### 3.3 Activity Monitoring and Cardiopulmonary Exercise Testing

The amount of vigorous activity was unchanged among the different time points (*p* = 0.36) and was therefore maintained during fasting by the exercise programme, without any sex (*p* = 0.47) or age (*p* = 0.41) differences (see Table S3). On the other hand, we observed changes in the breakdown of lower activity levels, with moderate and light activity levels decreasing during fasting (*p* < 0.001) and sedentary activity increasing (*p* < 0.002).

**FIGURE 4:**
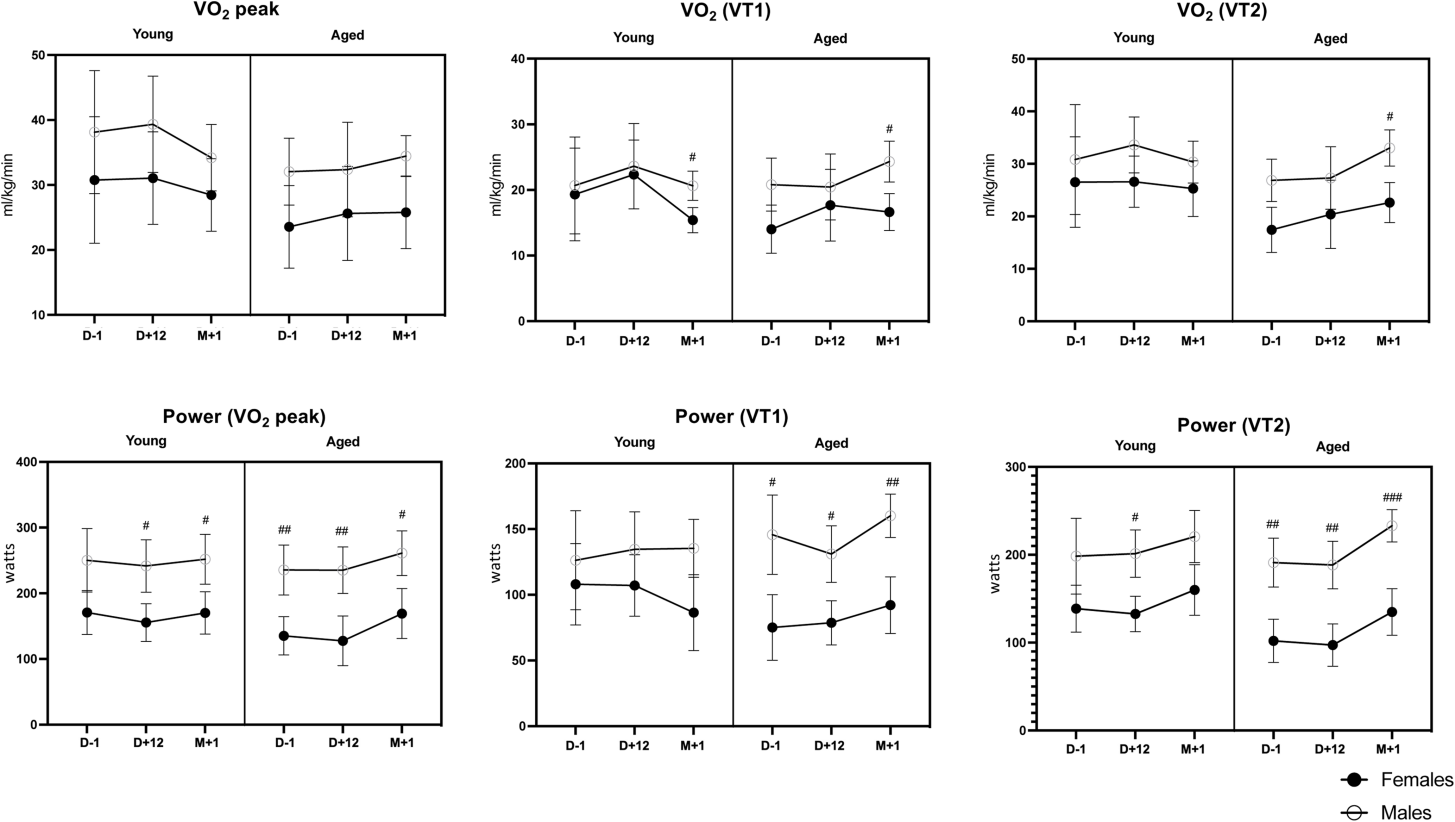
Cardiopulmonary exercise test results obtained before (D − 1), at the end (D + 12) and 1 month (M + 1) after the end of fasting, including oxygen uptake VO_2_ measures—top: maximum oxygen uptake capacity (VO_2_peak), VO_2_ achieved at the first and second ventilatory threshold (VT1 and VT2) and the corresponding ergometric power levels (graph data are mean ± 95% CI; post‐hoc comparisons between males and females; # = *p* < 0.05; ## = *p* < 0.01; ### = *p* < 0.001).

All details of peak exercise capacity testing and parameters are in Table S4 and Figure 4. Peak oxygen uptake (VO_2_peak) was remarkably stable over time (*p* = 0.669), without any decrease at D + 12 compared with baseline. There were no statistically significant two‐way interactions or age and sex effects. Ventilatory threshold (VT1 and VT2) changes over time relied on age, as illustrated by the significant time * age interactions (*p* = 0.026 and *p* = 0.042, respectively).

**FIGURE 5:**
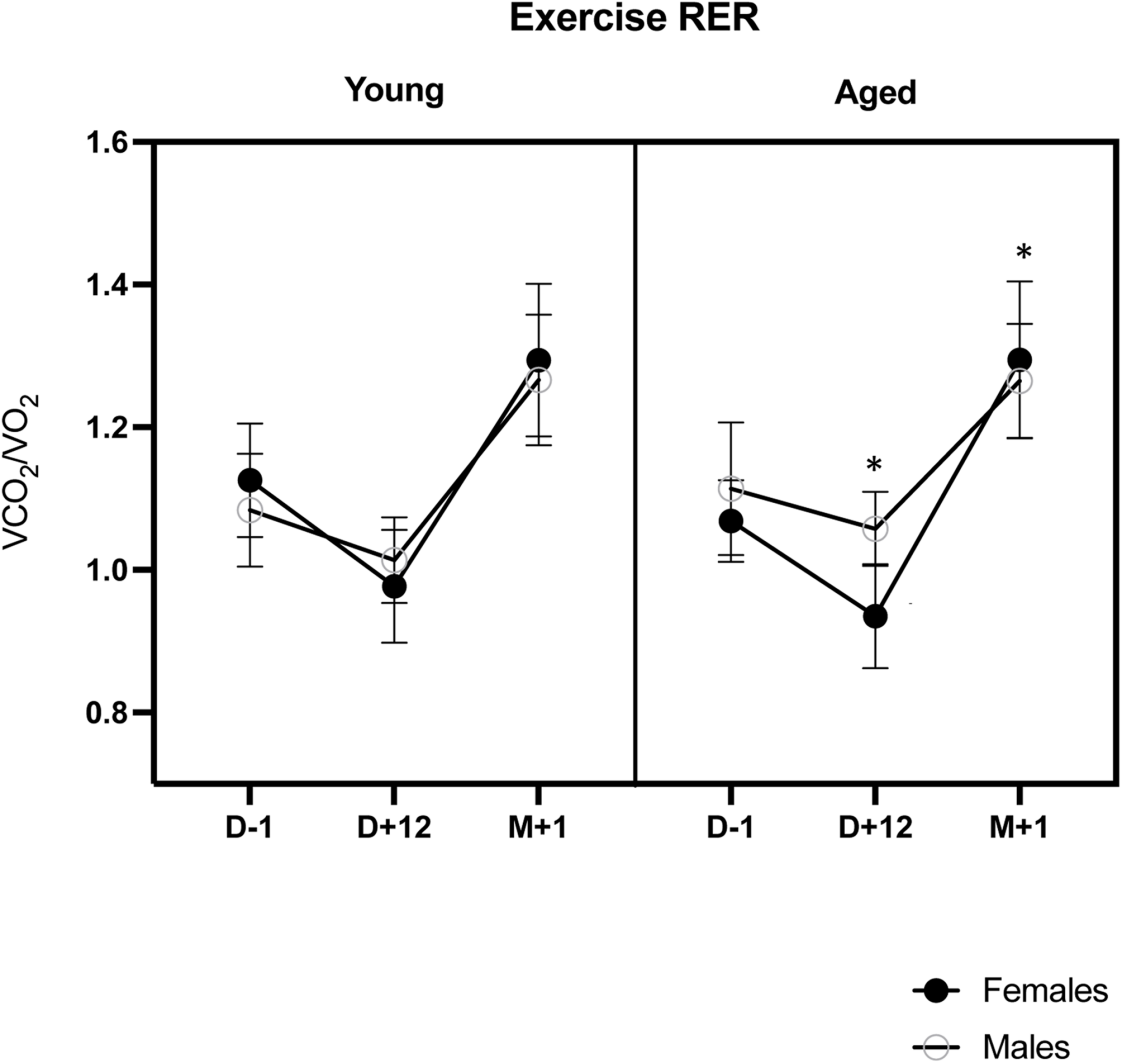
Exercise respiratory exchange ratio (RER) during cardiopulmonary exercise tests, before (D − 1), at the end (D + 12) and 1 month (M + 1) after the end of fasting (data are mean ± 95% CI; post‐hoc comparisons D + 12 and M + 1 vs. D − 1: * = *p* < 0.05).

The exercise respiratory exchange ratio (RER) varied significantly over time (*p* < 0.001), with a significant reduction at D + 12 and with a time * sex interaction (*p* = 0.017). Indeed, although males and females had similar RER ratios at baseline (*p* = 0.916) and M + 1 (*p* = 0.466), the RER ratio decreased significantly more at D + 12 in females (−12.7% from baseline) than in males (−5.7%) (*p* = 0.010) (see Figure 5).

### 3.4 MR Relaxometry, ^1^H‐MRS and Tissues Fat Composition on the Thigh

Table S5 provides all the details regarding the changes in skeletal muscle, SAT, and BMAT composition at the thigh level.

**FIGURE 6:**
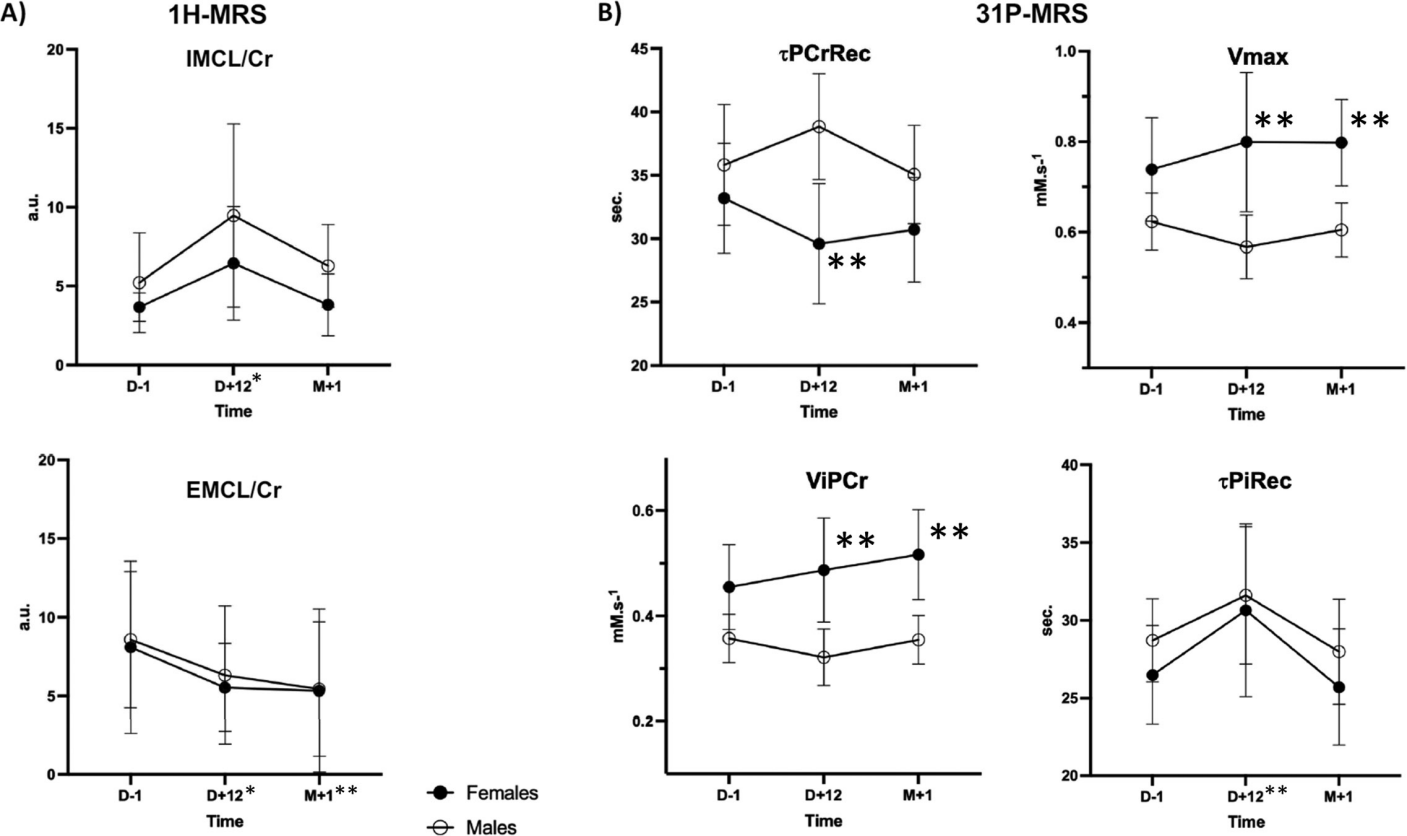
(A) Intramyocellular lipids (IMCL) and extramyocellular lipids (EMCL) content changes using ^1^H‐MRS before (D − 1), at the end (D + 12) and 1 month (M + 1) after the end of fasting; (B) mitochondrial oxidative capacity estimates using ^31^P‐MRS before (D − 1), at the end (D + 12) and 1 month (M + 1) after the end of fasting. Data are mean ± 95% CI; post‐hoc comparisons D + 12 and M + 1 versus D − 1: * = *p* < 0.05; ** = *p* < 0.01; male versus female: # = *p* < 0.05; ## = *p* < 0.01.

In skeletal muscle, relaxometry measures showed no changes in T2 but a significant T2* increase over time (*p* < 0.001) ceiling at D + 12, together with significant differences related to age (*p* = 0.015). In parallel, fat fraction (PDFF%) in muscle increased at the end of fasting (*p* = 0.002) and more significantly in older individuals (+22% at D + 12) (*p* = 0.019). ^1^H‐MRS provides further understanding of muscle content changes: IMCL exhibited time variations (*p* = 0.009), with a +113% average increase at the end of fasting (*p* = 0.019), and returned to baseline at M + 1. At the same time, EMCL decreased at D + 12 (*p* = 0.012, −28%) while remaining significantly lower at M + 1 than at baseline (*p* < 0.001) (see Figure 6A). There were no significant changes in taurine and choline metabolites in muscle tissue.

In SAT, T2* and PDFF both decreased during time (both *p* < 0.001), with a sex * time interaction and PDFF lowering more in males and at M + 1 (*p* = 0.005). The fat composition also changed until M + 1 with more SFA, and less MUFA and PUFA (*p* < 0.001) and sex differences (*p* < 0.001). In BMAT, there were no T2* and PDFF changes over time, with more transient changes in all fat composition (all *p* < 0.05).

### 3.5 Dynamic ^31^P‐MRS on the Calf Muscle

Table S6 provides the main variables quantified by dynamic ^31^P‐MRS (rest, during exercise and recovery) assessing the muscular energy balance at the different time points of the study (see also Table S7 for additional results). There were no significant differences in high‐energy phosphate intermediates, [PCr] and [Pi] remaining stable over time. The pH was significantly lowered at the end of fasting, existing at rest (*p* < 0.001), but acidosis did not worsen during exercise (*p* = 0.002) or recovery. Of note, there were sex differences with lower pH in females at rest (*p* = 0.015) and recovery (*p* = 0.010) pre‐existing at baseline. The PCr depletion/consumption during aerobic exercise was comparable between the fed and unfed phases and without differences across sex and age groups. Overall, mitochondrial oxidative capacity remained stable over time, with sex‐related differences (*p* = 0.022) and a faster recovery in females, especially at end‐fasting (*p* = 0.004) (Figure 6B). Note that τPiRec showed changes over time and increased at end‐fasting (*p* = 0.007).

## 4 Discussion

The acceptability of therapeutic fasting by conventional medicine has been notably hindered by concerns about its impact on skeletal muscle, questioning muscle loss and, ultimately, sarcopenia, implying that older subjects would be more exposed to this risk. Nevertheless, this has never been scientifically investigated in detail. Our study showed that, although muscle volume decreased to some extent after 12 days of fasting, skeletal muscle metabolism and function were maintained without any harmful effects even in older subjects. Importantly, these outcomes remained consistent even 1 month after fasting, whereas muscle volume restoration was underway.

### 4.1 Muscular Volume and Strength Measurements

The total skeletal muscle volume loss of the lower limbs at D + 12 (end‐fasting) of 542 mL (−5.4%) in the whole population should be put in perspective with the expected changes directly related to glycogen storage depletion. Such changes occur within a few days during fasting and dieting.

In sedentary untrained subjects adhering to a regular mixed diet, the average skeletal muscle glycogen storage is 1%–2% of wet muscle weight. However, factors such as diet and activity can elevate this percentage to 4%–5% in highly active individuals [34, 35]. Additionally, every gram of glycogen is bound to an estimated 3–4 g of water because of direct molecular bounding and osmosis [36, 37]. Considering a theoretical glycogen content of 1% at baseline, the calculated volume loss related to total glycogen depletion and its bounded water (3%–4%) would be 404–505 mL, close to the volume loss quantified in our study. Glycogen depletion experiments in nonathletes with muscle biopsies have shown that a single moderate‐intensity cycling exercise (~60% VO_2_max) of 60 min induced a 60%–70% muscle glycogen depletion [38], whereas the addition of a high‐intensity set of exercises is needed to reach an additional 20% muscle glycogen depletion corresponding to glycogen stored in fast‐twitch fibres [39]. Nevertheless, fast‐twitch fibres are more susceptible to glycogen depletion during fasting or caloric restriction [40]. Because our subjects spent 158(41) minutes of vigorous activity during fasting (Table S3), we can reasonably assume that most of the glycogen content was depleted at the end of fasting.

Therefore, the estimated volume loss corresponding to the combination of glycogen depletion and bounded water volume in the skeletal muscle of the lower limbs is close to the volume loss quantified in our study. This indirect assessment suggests that the actual protein loss in skeletal muscle related to neoglucogenesis is rather limited, as suggested by Laurens et al. [4].

An essential finding of the study is the total preservation of the lower limb's muscle function quantified by the MVC measures at the end of the fasting period (D + 12). This observation indicates that the high‐intensity anaerobic alactic metabolism pathway mainly related to fast‐twitch fibre recruitment is operational and intact. It also suggests the absence of impairment in the neural central nervous system output or central fatigue at the end of fasting (Reference S1).

### 4.2 Cardiopulmonary Exercise Testing

The maximal cardiopulmonary exercise ramped testing was used to quantify the overall ability to deliver oxygen to exercising skeletal muscles and the ability of the exercising muscles to extract oxygen. This latter point is addressed explicitly by ^31^P‐MRS measures discussed below.

Our study found that the peak oxygen uptake capacity (i.e., VO_2_peak) stayed remarkably stable over time and was maintained at the end of the fasting period, whereas the maximum power output at peak exercise was also maintained.

We also documented an improvement of the first ventilatory threshold (VT1), which is of practical interest from a functional point of view. From a physiological exercise standpoint, VT1 is associated with the initial rise and commencement of blood lactate accumulation. However, for nonathlete sedentary or active subjects, this translates to a better ability to achieve daily tasks, including walking, that are, for the majority, performed below submaximal exercise level. Therefore, increasing this VT1 would allow one to perform everyday activities at a lower relative level of the maximal level, thus limiting the impact on fatigue (Reference S2). The last important finding is observing of a decrease in the RER at the end of fasting. A decrease in the RER during an exercise test reflects a greater reliance on fat oxidation and utilization of plasma‐free fatty acids for energy production while signalling (if needed) the depletion of muscular carbohydrate stores (References S3 and S4). Although we found no difference in the response between age groups, females had lower RER than males, but only after fasting. Although most studies comparing substrate oxidation among genders describe lower RER in females, Tarnopolsky discussed that a lack of difference might be related to confounding factors (diet, body composition, time since last exercise boot, level of fitness, menstrual phase or amenorrhea) (Reference S5). Therefore, if the lack of difference in RER at baseline between males and females in our study is to be attributed, at least partially, to hormonal factors, it is worth noting that fasting unmasked the expected lower RER in females.

From a metabolic and substrate availability perspective, all these findings are very similar to those observed in endurance athletes practicing a ketogenic diet (KD). This diet is an increasingly popular conditioning strategy during endurance training in athletes to activate fat metabolism pathways, increase fat utilization as a primary muscle fuel while achieving very‐low glycogen stores and expose the body to circulating ketones (References S6 and S7). Although scientists keep exploring the benefits and drawbacks of KD as an ergogenic strategy, several studies have investigated its impact on endurance performance. After a 3‐week KD, two studies (References S8 and S9) showed significant increases in V02max, whereas three others found only preservation of VO_2_max (References S10–S12). All studies reported a shift in the RER ratio, illustrating the greater reliance on fat oxidation in ketosis.

### 4.3 MR Relaxometry, ^1^H‐MRS and Tissues Fat Composition on the Thigh

In skeletal muscle, PDFF measures, representing the overall lipid content, increased significantly during fasting and returned to baseline at M + 1.

Skeletal muscle T2 relaxation times remained nearly stable at the end‐fasting. This measure is known to be sensitive to both free water content and, to a lesser degree, lipid content, which can theoretically counteract each other (References S13–S15). T2*, on the other hand, increased markedly at end‐fasting but returned to baseline values at M + 1. T2* is notably influenced by the size of lipid droplets (LDs). In skeletal muscle and liver, smaller cytoplasmic LDs have longer T2* values than larger ones. T2* increases because smaller LDs have a higher surface‐to‐volume ratio, allowing more contact between lipid‐associated and mobile water protons (Reference S16). The relatively greater surface area of smaller LDs also translates into an increased interface for lipid metabolic enzymes and potentially a greater capacity to mobilize lipids (Reference S17). Additionally, LD size can adapt and decrease within minutes during energy stress conditions such as exercise (Reference S18). As lipids represent the primary energy substrate, stored triacylglycerols (TAGs) in LD undergo increased delivery to mitochondria. The enhanced beta‐oxidation rate and fatty acid trafficking lead to LD shrinking in muscles because of increased demand (Reference S19). During nutrient stress conditions such as during fasting, increased FA trafficking involves several mechanisms that are codependent on LD lipolysis, lipophagy and mitochondrial fusion dynamics (References S20 and S21). When increased accessibility of lipid fuels is needed, LD configuration changes with more numerous but smaller LDs, helping a rapid supply of fat to mitochondria, whereas LDs are predominantly located very close or in direct contact with mitochondria (References S19 and S22).

^1^H‐MRS in muscles enables the localization and quantification of lipids in tissues and showed a significant increase of intracellular lipids (IMCL) at D + 12. In contrast, the extracellular lipids pool (EMCL) lowered significantly. This lipid redistribution in muscle favouring intracellular lipid components occurs within the first days of fasting, as described after a 3‐day (Reference S23) and 5‐day fast (Reference S24). Although none of these studies addressed the dynamics after food reintroduction, we showed that IMCL returned to prefasting levels. At the same time, EMCL remained below prefasting levels, with a muscle fat content measured by PDFF below baseline values. This dynamic clearly illustrates the adaptive reciprocity of lipid and glucose metabolism, which switches from one system to another (Reference S25).

In both BMAT and SAT tissues, the proportions of PUFAs and MUFAs decreased and were mobilized during fasting. Mobilization of FAs has been demonstrated to be selective, depending on chain length, unsaturation and isomerism (References S26 and S27). The mobilization selectivity is linked to the dispersion of TAGs within lipid droplets depending on their polarity. The shorter and less saturated FAs, which are more polar, are positioned on the outer edge of the lipid droplet. This arrangement allows them to be more easily targeted by hormone‐sensitive lipases, thereby safeguarding the longer‐chain FAs (Reference S28).

Taurine is the most abundant free amino acid in skeletal muscle, implicated in critical cellular processes including substrate regulation (uptake, storage and oxidation), the excitation‐contraction processes, the cellular osmoregulation and has antioxidant activity (References S29 and S30). Using ^1^H‐MRS measures, we showed that long‐term fasting led to conserving muscle taurine content. Although acute conditions such as exercise have led to decreased taurine concentration in rats (Reference S31), our results are consistent with other experimental data showing taurine conservation under starvation conditions involving the cysteine and coenzyme‐A pathways and taurine biosynthesis by the liver (Reference S32). Choline is a metabolite that affects skeletal muscle by modulating fat and protein metabolism, inflammation, apoptosis and autophagy (References S33 and S34). Although choline endogenous synthesis is insufficient to support human choline requirements (Reference S35), we showed that 12‐day fasting did not affect choline skeletal muscle content.

### 4.4 Dynamic ^31^P‐MRS on the Calf Muscle

Dynamic ^31^P‐MRS enabled us to repeatedly and noninvasively measure variations in high‐energy phosphorylated metabolites in a calibrated and submaximum exercise, thereby avoiding glycolytic metabolism. The kinetics of post‐exercise recovery of PCr provided unique information regarding mitochondrial oxidative capacity [27] (References S36 and S37). Overall, all time constants, τPCrRec (the rate of PCr resynthesis), ViPCr (maximal mitochondrial ATP production), and Vmax (maximal efficiency of oxidative phosphorylation, ~mitochondrial density) remained preserved after long‐term fasting. More interesting is the finding that females improved their mitochondrial function significantly (lower τPCrRec, higher ViPCr and Vmax) not only compared with males but also from their baseline value at the end of fasting. This improvement persisted 1 month after food reintroduction. This finding is coherent with the lower RER measured during cardiopulmonary exercise testing and sheds light on the greater capability of fat oxidation in females.

Several gender‐related physiological differences explain the improved capabilities of fat oxidation in females (Reference S38). Females have a more significant proportion of slow‐twitch fibres with greater vasodilatory capacity and capillarization (Reference S39). Oestrogen has been shown to upregulate genes involved in fat metabolism, including lipoprotein lipase. Substrate utilization is improved because of greater mRNA expression of genes associated with fatty acid metabolism, including CD36 (Reference S40), and the presence of oestrogen‐mediated reserves of IMCL supports the fuel demands with a more significant percentage of IMCL in contact with mitochondria when compared with males (Reference S41). In addition, Montero et al. (Reference S42) showed that improved fat oxidation during exercise is underpinned by augmented mitochondrial volume density and better fatty acid and lactate oxidative capacity of skeletal muscle. Earlier studies have been conducted during 2‐ to 5‐day fasting experiments during near‐maximal or maximal exercises that lead to exercise‐induced acidosis and lactate release (References S43–S45). A minimal pH lowering was observed at D + 12 compared with baseline but did not impact exercise because pH remained stable at the end of the exercise. This minimal pH difference may be because of the balance in physiological ketosis between acetoacetate, acetone, and beta‐hydroxybutyrate (Reference S46). The kinetics of Pi recovery (τPiRec) showed a significant difference, with a longer return to the basal state. Unlike the dynamics of PCr recovery, Pi recovery is not only because of mitochondrial respiration but is dependent on other factors, including pH buffering capacity (Reference S47).

## 5 Limitations

We must acknowledge a few limitations. First, the limited sample size implies that the study findings may not be generalizable to the broader population of individuals. The coupling of age and gender features remains exploratory for the time being but may guide the design of future studies.

Also, the absence of a control group limits the ability to infer causality, making it difficult to separate the effects of fasting from other aspects of the intervention.

## 6 Conclusions

Our results indicate no disturbance of muscle metabolism caused by long‐term fasting, with no evidence of a negative impact on mitochondrial respiration or general muscle cell function. The recorded muscle utilization, although not negligible, does not affect muscle strength and healthy human muscle performance in anaerobic lactic efforts. Our results also demonstrate an adaptation of the mitochondrial respiration supply chain with lipid trafficking from the extracellular to the intracellular compartment. This adaptation is coherent with the RER decrease, indicating a shift in substrate utilization, with a preference for lipids after the fasting period, showcasing the harmlessness of fasting for muscle metabolism. This preservation of muscle function during fasting suggests that humans, like wild animals, were evolutionary adapted by seasonal food shortages to fast periodically. This further indicates that there is no muscular contraindication when using this approach.

This adaptive phenomenon is likely geared towards ensuring better availability of fatty acids, which become the primary energetic substrate of the mitochondrial respiratory chain. Because the diverse intracellular compartments contribute to global cell homeostasis and the intracellular trafficking of lipids and proteins are closely related, these results open new perspectives while warranting further investigations. Of crucial interest is the improvement of the first ventilatory threshold, which, from a functional point of view, directly impacts everyday activities performed at a lower relative level of the maximal level, with immediate consequences on perceived fatigue.

## Authors Contributions

Pierre Croisille, Magalie Viallon, Robin Mesnage, Franziska Grundler and Françoise Wilhelmi de Toledo conceived and conceptualized the study. Pierre Croisille and Magalie Viallon acquired the MR data and were responsible of the MR protocols. Franziska Grundler was responsible for the project administration and blood data collection. Antoine Naegel and Hélène Ratiney analysed all the MRS data. Thu Nguyen, Thomas Grenier, Benjamin Leporq and Magalie Viallon joined efforts for the analysis of the MRI muscle data. Pierre Croisille, Antoine Naegel and Magalie Viallon realized and/or verified the statistical analyses of the data. All authors actively participated to the manuscript editing and reviewing.

## Conflicts of Interest

Antoine Naegel, Magalie Viallon, Helène Ratiney, Thu Nguyen, Benjamin Leporq, Djahid Kennouche, Thomas Grenier, Jean‐Michel Guy and Pierre Croisille declare that they have no conflict of interest. Franziska Grundler, Robin Mesnage and Françoise Wilhelmi de Toledo are employed by Buchinger Wilhelmi Development & Holding GmbH in Uberlingen. Robin Schulze declares no conflict of interest.

## Supporting information


**Data S1** Supporting information

**Table S1** Main parameters of the MR sequences.

**Table S2** Baseline demographic data of subjects enrolled in the GENESIS study.

**Table S3** Actimetry counts.

**Table S4** Cardiopulmonary exercise testing summary results (mean (SD), overall, and stratified by sex and age). The table presents the effect of time, sex, and age using a repeated‐measures mixed‐effects model. When applicable, pairwise post‐hoc comparisons used a Tukey test to correct for multiple comparisons. For all analyses, significance was accepted at *p* < 0.05. (Legend for post‐hoc comparisons: D + 12 & D + 30 vs D‐1: * = p < 0.05; ** = *p* < 0.01; *** = *p* < 0.001. For Sex and Age variable, post‐hoc comparisons are for each time point).

**Table S5** Relaxometry and **thigh** fat composition: 1H‐MRS results (mean (SD), overall, and stratified by sex and age). Analysed tissues are tight muscle, subcutaneous adipose tissue (SAT), and bone marrow adipose tissue (BMAT). The table presents the effect of time, sex, and age, using a repeated‐measures mixed‐effects model. When applicable, pairwise post‐hoc comparisons used a Tukey test to correct for multiple comparisons. For all analyses, significance was accepted at *p* < 0.05. (Legends for post‐hoc comparisons: comparisons are against D‐1 used as a control for time variable: * = p < 0.05; ** = *p* < 0.01; *** = *p* < 0.001. For Sex and Age variable, post‐hoc comparisons are for each time point.)

**Table S6** 31P‐MRS findings at rest, during exercise, and recovery (mean (SD), overall, and stratified by sex and age). The table presents the effect of time, sex, and age, using a repeated‐measures mixed‐effects model. When applicable, pairwise post‐hoc comparisons used a Tukey test to correct for multiple comparisons. For all analyses, significance was accepted at *p* < 0.05. (Legends for post‐hoc comparisons: for time variable, comparisons are against D‐1 used as a control: * = p < 0.05; ** = *p* < 0.01; *** = *p* < 0.001. For Sex and Age variable, post‐hoc comparisons are for each time point.)

**Table S7** Full list of 31P‐MRS quantified biomarkers

**Figure S1** Supporting information

**Figure S2A** Supporting information

**Figure S2B** Supporting information
