# Supplementary material for: Impact of Long‐Term Fasting on Skeletal Muscle: Structure, Energy Metabolism and Function Using 31P/1H MRS and MRI
Source: J Cachexia Sarcopenia Muscle. 2025 Apr 11;16(2):e13773. doi: 10.1002/jcsm.13773 (PMC11986369; doi:10.1002/jcsm.13773)
Supplement: Supplementary file 1 — Data S1 Supporting information [file JCSM-16-e13773-s011.docx]

**Supplementary materials**

***Materials and Methods***

***Activity monitoring***

To quantify the physical activity during the experiment, we asked the participants to wear a wrist-worn accelerometer (MotionWatch 8©, CamNtech, Cambridge, UK) continuously, starting two weeks before fasting, continuing throughout the fasting period, and concluding two weeks after re-feeding. MotionWatch 8^©^ is an unobtrusive, waterproof, wrist-worn device containing a light sensor and a tri-axial accelerometer detecting acceleration in a 0.01–8 g range. The device was worn on the non-dominant wrist, recording activity counts and light intensity using 60-s epochs. To achieve representative estimates of the daily activity reports, the measurements were accumulated over ten days at baseline, during fasting, and post-fasting period^s48^. The cut-points used for activity level calibration (sedentary, light, moderate, or vigorous activity levels) were those established in non-athlete older healthy adults by Landry et al.^s49^.

***Muscular strength measurements***

The quadricipital maximal voluntary contraction (MVC) was measured on a dynamometer (ARS dynamometry; SP2, Ltd., Ljubljana, Slovenia) with subjects in an upright sitting position performing isometric knee extensor contractions. A brief warm-up consisting of 5–7 submaximal contractions of the quadriceps at a progressively increasing intensity of up to 90% of perceived maximal strength was subsequently performed. Calf MVC measurements were performed using the MR-compatible ergometer (ErgoSpect GmbH, Innsbruck, Austria) with subjects lying supine in MR scanner. In each case, participants were instructed to exert and maintain maximum strength for 3 seconds through verbal stimuli. Participants then performed 3 MVCs, with the peak value taken to calculate submaximal forces.

***Cardiopulmonary exercise testing***

A maximal cardiopulmonary exercise cycle ergometer test was performed using a ramped incremental protocol with resistance increased progressively by 15W.min^-1^ increments (1 W every 4s.). Patients were seated on a bike (Monark Ergomedic 839E, Vansbro, Sweden) with 12-lead electrocardiogram and gas exchange monitoring (Vyntus CPX, CareFusion, Hoechberg, Germany). Patients started with a 2-minute warm-up period at 25 to 50 W load; then, resistance increased linearly until maximum exercise capacity was reached (i.e., VO_2_peak) and voluntary exhaustion occurred. Patients were instructed to keep a cadence near 80 revolutions (rev).min^-1^ and were encouraged during the protocol. At exhaustion, patients were monitored until cardiorespiratory parameters returned to baseline levels.

***Supplementary references***

s1. Place N, Yamada T, Bruton JD, Westerblad H. Muscle fatigue: from observations in humans to underlying mechanisms studied in intact single muscle fibres. *Eur J Appl Physiol* 2010;**110**:1–15.

s2. Brownstein CG, Twomey R, Temesi J, Medysky ME, Culos-Reed SN, Millet GY. Mechanisms of Neuromuscular Fatigability in People with Cancer-Related Fatigue. *Medicine & Science in Sports & Exercise* 2022;**54**:1355–1363.

s3. Achten J, Gleeson M, Jeukendrup AE. Determination of the exercise intensity that elicits maximal fat oxidation. *Med Sci Sports Exerc* 2002;**34**:92–97.

s4. Jeukendrup AE, Wallis GA. Measurement of substrate oxidation during exercise by means of gas exchange measurements. *Int J Sports Med* 2005;**26 Suppl 1**:S28-37.

s5. Tarnopolsky MA. Sex differences in exercise metabolism and the role of 17-beta estradiol. *Med Sci Sports Exerc* 2008;**40**:648–654.

s6. Volek JS, Noakes T, Phinney SD. Rethinking fat as a fuel for endurance exercise. *Eur J Sport Sci* 2015;**15**:13–20.

s7. Noakes T, Volek JS, Phinney SD. Low-carbohydrate diets for athletes: what evidence? *Br J Sports Med* 2014;**48**:1077–1078.

s8. Burke LM, Ross ML, Garvican-Lewis LA, Welvaert M, Heikura IA, Forbes SG *et al.* Low carbohydrate, high fat diet impairs exercise economy and negates the performance benefit from intensified training in elite race walkers. *J Physiol* 2017;**595**:2785–2807.

s9. Carr AJ, Sharma AP, Ross ML, Welvaert M, Slater GJ, Burke LM. Chronic Ketogenic Low Carbohydrate High Fat Diet Has Minimal Effects on Acid–Base Status in Elite Athletes. *Nutrients* 2018;**10**:236–249.

s10. Shaw DM, Merien F, Braakhuis A, Maunder ED, Dulson DK. Effect of a Ketogenic Diet on Submaximal Exercise Capacity and Efficiency in Runners. *Med Sci Sports Exerc* 2019;**51**:2135–2146.

s11. Phinney SD, Bistrian BR, Evans WJ, Gervino E, Blackburn GL. The human metabolic response to chronic ketosis without caloric restriction: preservation of submaximal exercise capability with reduced carbohydrate oxidation. *Metabolism* 1983;**32**:769–776.

s12. McSwiney FT, Wardrop B, Hyde PN, Lafountain RA, Volek JS, Doyle L. Keto-adaptation enhances exercise performance and body composition responses to training in endurance athletes. *Metabolism* 2018;**83**:e1–e2.

S13. Schwenzer NF, Martirosian P, Machann J, Schraml C, Steidle G, Claussen CD *et al.* Aging effects on human calf muscle properties assessed by MRI at 3 Tesla. *J Magn Reson Imaging* 2009;**29**:1346–1354.

s14. Varghese J, Scandling D, Joshi R, Aneja A, Craft J, Raman SV *et al.* Rapid assessment of quantitative *T* _1_ , *T* _2_ and *T* _2_ * in lower extremity muscles in response to maximal treadmill exercise: Mr Relaxometry In Skeletal Muscle. *NMR Biomed* 2015;**28**:998–1008.

s15. Cameron IL, Ord VA, Fullerton GD. Characterization of proton NMR relaxation times in normal and pathological tissues by correlation with other tissue parameters. *Magnetic Resonance Imaging* 1984;**2**:97–106.

s16. Weidlich D, Honecker J, Boehm C, Ruschke S, Junker D, Van AT *et al.* Lipid droplet-size mapping in human adipose tissue using a clinical 3T system. *Magn Reson Med* 2021;**86**:1256–1270.

s17. Bosma M. Lipid droplet dynamics in skeletal muscle. *Exp Cell Res* 2016;**340**:180–186.

s18. Alsted TJ, Ploug T, Prats C, Serup AK, Høeg L, Schjerling P *et al.* Contraction-induced lipolysis is not impaired by inhibition of hormone-sensitive lipase in skeletal muscle. *J Physiol* 2013;**591**:5141–5155.

s19. Schrauwen-Hinderling VB, Hesselink MKC, Schrauwen P, Kooi ME. Intramyocellular lipid content in human skeletal muscle. *Obesity (Silver Spring)* 2006;**14**:357–367.

s20. Shaw CS, Jones DA, Wagenmakers AJM. Network distribution of mitochondria and lipid droplets in human muscle fibres. *Histochem Cell Biol* 2008;**129**:65–72.

s21. Rambold AS, Cohen S, Lippincott-Schwartz J. Fatty acid trafficking in starved cells: regulation by lipid droplet lipolysis, autophagy, and mitochondrial fusion dynamics. *Dev Cell* 2015;**32**:678–692.

s22. Hoppeler H. Exercise-induced ultrastructural changes in skeletal muscle. *Int J Sports Med* 1986;**7**:187–204.

s23. Stannard SR, Thompson MW, Fairbairn K, Huard B, Sachinwalla T, Thompson CH. Fasting for 72 h increases intramyocellular lipid content in nondiabetic, physically fit men. *American Journal of Physiology-Endocrinology and Metabolism* 2002;**283**:E1185–E1191.

s24. Wietek BM, Machann J, Mader I, Thamer C, Häring H-U, Claussen CD *et al.* Muscle Type Dependent Increase in Intramyocellular Lipids during Prolonged Fasting of Human Subjects: A Proton MRS Study. *Horm Metab Res* 2004;**36**:639–644.

s25. Soeters MR, Soeters PB, Schooneman MG, Houten SM, Romijn JA. Adaptive reciprocity of lipid and glucose metabolism in human short-term starvation. *American Journal of Physiology-Endocrinology and Metabolism* 2012;**303**:E1397–E1407.

s26. Raclot T. Selective mobilization of fatty acids from adipose tissue triacylglycerols. *Prog Lipid Res* 2003;**42**:257–288.

s27. Mustonen A-M, Käkelä R, Käkelä A, Pyykönen T, Aho J, Nieminen P. Lipid metabolism in the adipose tissues of a carnivore, the raccoon dog, during prolonged fasting. *Exp Biol Med (Maywood)* 2007;**232**:58–69.

s28. Raclot T, Groscolas R. Differential mobilization of white adipose tissue fatty acids according to chain length, unsaturation, and positional isomerism. *J Lipid Res* 1993;**34**:1515–1526.

s29. Schaffer SW, Ju Jong C, KC R, Azuma J. Physiological roles of taurine in heart and muscle. *Journal of Biomedical Science* 2010;**17**:S2.

s30. Spriet LL, Whitfield J. Taurine and skeletal muscle function. *Curr Opin Clin Nutr Metab Care* 2015;**18**:96–101.

s31. Matsuzaki Y, Miyazaki T, Miyakawa S, Bouscarel B, Ikegami T, Tanaka N. Decreased taurine concentration in skeletal muscles after exercise for various durations. *Med Sci Sports Exerc* 2002;**34**:793–797.

s32. Serrano-Contreras JI, García-Pérez I, Meléndez-Camargo ME, Zepeda LG. NMR-Based Metabonomic Analysis of Physiological Responses to Starvation and Refeeding in the Rat. *J Proteome Res* 2016;**15**:3241–3254.

s33. da Costa K-A, Badea M, Fischer LM, Zeisel SH. Elevated serum creatine phosphokinase in choline-deficient humans: mechanistic studies in C2C12 mouse myoblasts. *Am J Clin Nutr* 2004;**80**:163–170.

s34. Ueland PM. Choline and betaine in health and disease. *Journal of inherited metabolic disease* 2011;**34**:3–15.

s35. Moretti A, Paoletta M, Liguori S, Bertone M, Toro G, Iolascon G. Choline: An Essential Nutrient for Skeletal Muscle. *Nutrients* 2020;**12**:2144.

s36. Jill M.Slade, Towse TF, DeLano MC, Wiseman RW, Meyer RA. A gated 31P NMR method for the estimation of phosphocreatine recovery time and contractile ATP cost in human muscle. *NMR Biomed* 2006;**19**:573–580.

s37. Kemp GJ. Muscle Studies by ^31^ P MRS. In: Harris RK, Wasylishen RL, editors. *eMagRes*. John Wiley & Sons, Ltd: Chichester, UK; 2015. pp. 525–534.

s38. Tiller NB, Elliott-Sale KJ, Knechtle B, Wilson PB, Roberts JD, Millet GY. Do Sex Differences in Physiology Confer a Female Advantage in Ultra-Endurance Sport? *Sports Med* 2021;**51**:895–915.

s39. Staron RS, Hagerman FC, Hikida RS, Murray TF, Hostler DP, Crill MT *et al.* Fiber type composition of the vastus lateralis muscle of young men and women. *J Histochem Cytochem* 2000;**48**:623–629.

s40. Kiens B, Roepstorff C, Glatz JFC, Bonen A, Schjerling P, Knudsen J *et al.* Lipid-binding proteins and lipoprotein lipase activity in human skeletal muscle: influence of physical activity and gender. *Journal of Applied Physiology* 2004;**97**:1209–1218.

s41. Devries MC. Sex-based differences in endurance exercise muscle metabolism: impact on exercise and nutritional strategies to optimize health and performance in women. *Exp Physiol* 2016;**101**:243–249.

s42. Montero D, Madsen K, Meinild-Lundby A-K, Edin F, Lundby C. Sexual dimorphism of substrate utilization: Differences in skeletal muscle mitochondrial volume density and function. *Exp Physiol* 2018;**103**:851–859.

s43. Phang PT, Swinamer DL, Eccles R, Lunt JA, Allen P, Jeejeebhoy K *et al.* Muscle function testing and 31P-NMR spectroscopy in fasted healthy volunteers. *Journal of Critical Care* 1987;**2**:156–161.

s44. Lunt JA, Allen PS, Brauer M, Swinamer D, Treiber EO, Belcastro A *et al.* An evaluation of the effect of fasting on the exercise-induced changes in pH and Pi/PCr from skeletal muscle. *Magnetic Resonance in Medicine* 1986;**3**:946–952.

s45. Miller RG, Carson PJ, Moussavi RS, Green A, Baker A, Boska MD *et al.* Factors which influence alterations of phosphates and pH in exercising human skeletal muscle: Measurement error, reproducibility, and effects of fasting, carbohydrate loading, and metabolic acidosis. *Muscle Nerve* 1995;**18**:60–67.

s46. Laffel L. Ketone bodies: a review of physiology, pathophysiology and application of monitoring to diabetes. *Diabetes Metab Res Rev* 1999;**15**:412–426.

s47. Korzeniewski B. Regulation of oxidative phosphorylation in different muscles and various experimental conditions. *Biochem J* 2003;**375**:799–804.

s48. Resnick B, Boltz M, Galik E, Fix S, Zhu S. Feasibility, Reliability, and Validity of the MotionWatch 8 to Evaluate Physical Activity Among Older Adults With and Without Cognitive Impairment in Assisted Living Settings. Journal of Aging and Physical Activity 2021;29:391–399.

s49. Landry GJ, Falck RS, Beets MW, Liu-Ambrose T. Measuring physical activity in older adults: calibrating cut-points for the MotionWatch 8(©). Front Aging Neurosci 2015;7:165.

***Supplementary tables legends***

Figure S1: 1H-MRS and 31P-MRS dynamic acquisitions: A) 3 spectra (D-1, D+12, and M+1 after the end of fasting), showing IMCL and EMCL resonance variations in a 34 y.o. female. B) Single-voxel STEAM 1H-MRS acquisitions placed in the vastus medialis quadriceps’ head. C) Typical 31P spectrum with highlighted main peaks of metabolites of interest. D) Placement of saturation bands (yellow stripe) and shim box (green) on muscles used for MRS 31P acquisition during dynamic exercise.

Figure S2: A) Workflow with the different steps ensuring the reproducibility of muscle volume measurements using Dixon 3D GRE anatomical sequences from initial image planning to volume segmentation; B) Workflow with the different steps ensuring the reproducibility of the CSE 3D axial measurements: central slice identification and image planning.

**Table S1**: Main parameters of the MR sequences.

**Table S2**: Baseline demographic data of subjects enrolled in the GENESIS study.

**Table S3**: Actimetry counts.

**Table S4**: Cardiopulmonary exercise testing summary results (mean (SD), overall, and stratified by sex and age). The table presents the effect of time, sex, and age using a repeated-measures mixed-effects model. When applicable, pairwise post-hoc comparisons used a Tukey test to correct for multiple comparisons. For all analyses, significance was accepted at p< 0.05. (Legend for post-hoc comparisons: D+12 & D+30 vs D-1 : *=p<0.05; **=p<0.01; ***=p<0.001. For Sex and Age variable, post-hoc comparisons are for each time point).

**Table S5**: Relaxometry and **thigh** fat composition: 1H-MRS results (mean (SD), overall, and stratified by sex and age). Analyzed tissues are tight muscle, subcutaneous adipose tissue (SAT), and bone marrow adipose tissue (BMAT). The table presents the effect of time, sex, and age, using a repeated-measures mixed-effects model. When applicable, pairwise post-hoc comparisons used a Tukey test to correct for multiple comparisons. For all analyses, significance was accepted at p< 0.05. (Legends for post-hoc comparisons: comparisons are against D-1 used as a control for time variable: *=p<0.05; **=p<0.01; ***=p<0.001. For Sex and Age variable, post-hoc comparisons are for each time point.)

**Table S6**: 31P-MRS findings at rest, during exercise, and recovery (mean (SD), overall, and stratified by sex and age). The table presents the effect of time, sex, and age, using a repeated-measures mixed-effects model. When applicable, pairwise post-hoc comparisons used a Tukey test to correct for multiple comparisons. For all analyses, significance was accepted at p< 0.05. (Legends for post-hoc comparisons: for time variable, comparisons are against D-1 used as a control: *=p<0.05; **=p<0.01; ***=p<0.001. For Sex and Age variable, post-hoc comparisons are for each time point.)

**Table S7**: Full list of 31P-MRS quantified biomarkers
