# Supplementary material for: Impact of Long‐Term Fasting on Skeletal Muscle: Structure, Energy Metabolism and Function Using 31P/1H MRS and MRI
Source: J Cachexia Sarcopenia Muscle. 2025 Apr 11;16(2):e13773. doi: 10.1002/jcsm.13773 (PMC11986369; doi:10.1002/jcsm.13773)
Supplement: Supplementary file 2 — Table S1 Main parameters of the MR sequences. [file JCSM-16-e13773-s004.docx]

Table S1

| Parameters | CSE-MRI | Dixon-3D-GRE |
| --- | --- | --- |
| Sequence Type | 3D Multi-echo-gradient-echo | 3D Multi-echo-gradient-echo |
| Number of echoes | 10 | 2 |
| Acquisition Plane | Transversal | Coronal |
| FOV | 400*250 | 500*312 |
| Slice Thickness (mm) | 5 | 1 |
| Acq. Pixel size (mm^2^) | 1. 3 * 1.3 * 2 | 1.2 * 1.2 * 1.2 |
| Matrix size | 160*128 | 260*416 |
| Repetition time (msec) | 15.50 | 4.08 |
| Echo time (msec) | 12 echos 1..2/14.40 | 1.29/2.52 |
| Flip Angle(°) | 5° | 9° |
| Bandwidth(Hz/Px) | 1560 | 1000 |
| Averages | 1 | 1 |
| Fat suppression | None | Dixon |
| Partial Fourier | 7/8 | 7/8 |
| Total Acq. Time (min) | 2:08 | 5:16 |

| Parameters | 1H-MRS | 31P-MRS |
| --- | --- | --- |
| Sequence Type | Single Voxel STEAM | FID |
| Number of samples | 2048 | 512 |
| Acq. Pixel size (mm^2^) | 30*30*30 | - |
| Voxel size (mm3) | 27000 | - |
| Nex/ Averages | 180 | - |
| Repetition time (msec) | 2000 | 4 |
| Echo time (msec) | 20 | 0.35 |
| Flip Angle(°) | 90 | 90 |
| Bandwidth(Hz) | 3000 | 2500 |
| Water suppression | yes | none |
