# Supplementary material for: Impact of Long‐Term Fasting on Skeletal Muscle: Structure, Energy Metabolism and Function Using 31P/1H MRS and MRI
Source: J Cachexia Sarcopenia Muscle. 2025 Apr 11;16(2):e13773. doi: 10.1002/jcsm.13773 (PMC11986369; doi:10.1002/jcsm.13773)
Supplement: Supplementary file 3 — Table S2 Baseline demographic data of subjects enrolled in the GENESIS study. [file JCSM-16-e13773-s005.docx]

Table S2

| sex | age group | | N subjects | | age (years) | height (cm) | Weight (kg) | BMI (kg/m2) |
| --- | --- | --- | --- | --- | --- | --- | --- | --- |
| Female | young (<50) | | 8 | | 38 (7.45) | 168.33 (5.2) | 71.88 (9.65) | 25.41 (3.59) |
| Female | aged (≥50) | | 8 | | 60.25 (7.01) | 165.88 (5.62) | 73.8 (10.33) | 26.96 (4.65) |
| Male | young (<50) | | 8 | | 38.25 (5.47) | 182.88 (5.3) | 84.33 (17.16) | 25.12 (4.52) |
| Male | aged (≥50) | | 8 | | 57.13 (4.94) | 178.88 (6.06) | 86.15 (8.47) | 26.98 (3.04) |
| Total | |  | | 32 | 48.09 (12.11) | 173.82 (8.89) | 78.82 (12.95) | 26.1 (3.9) |
