# Supplementary material for: Impact of Long‐Term Fasting on Skeletal Muscle: Structure, Energy Metabolism and Function Using 31P/1H MRS and MRI
Source: J Cachexia Sarcopenia Muscle. 2025 Apr 11;16(2):e13773. doi: 10.1002/jcsm.13773 (PMC11986369; doi:10.1002/jcsm.13773)
Supplement: Supplementary file 4 — Table S3 Actimetry counts. [file JCSM-16-e13773-s002.docx]

Table S3

| **Activity level category** | **D-1** | **D+12** | **M+1** |
| --- | --- | --- | --- |
| Vigorous (min) | 168 (56.31) | 158.73 (41.66) | 171.79(52.82) |
| Moderate (min) | 153.63 (30.19) | 132.32 (18.87) | 164.17 (22.66) |
| Moderate-to-Vigorous (min) | 321.62 (72.37) | 291.05 (44.88) | 335.97 (58.2) |
| Low (min) | 240.28 (46.32) | 209.74 (40.92) | 250.49 (46.84) |
| Sedentary (min) | 384.02 (67.12) | 466.02 (46.79) | 409.16 (93.5) |
