# Supplementary figures and images for: Impact of Long‐Term Fasting on Skeletal Muscle: Structure, Energy Metabolism and Function Using 31P/1H MRS and MRI
Source: J Cachexia Sarcopenia Muscle. 2025 Apr 11;16(2):e13773. doi: 10.1002/jcsm.13773 (PMC11986369; doi:10.1002/jcsm.13773)

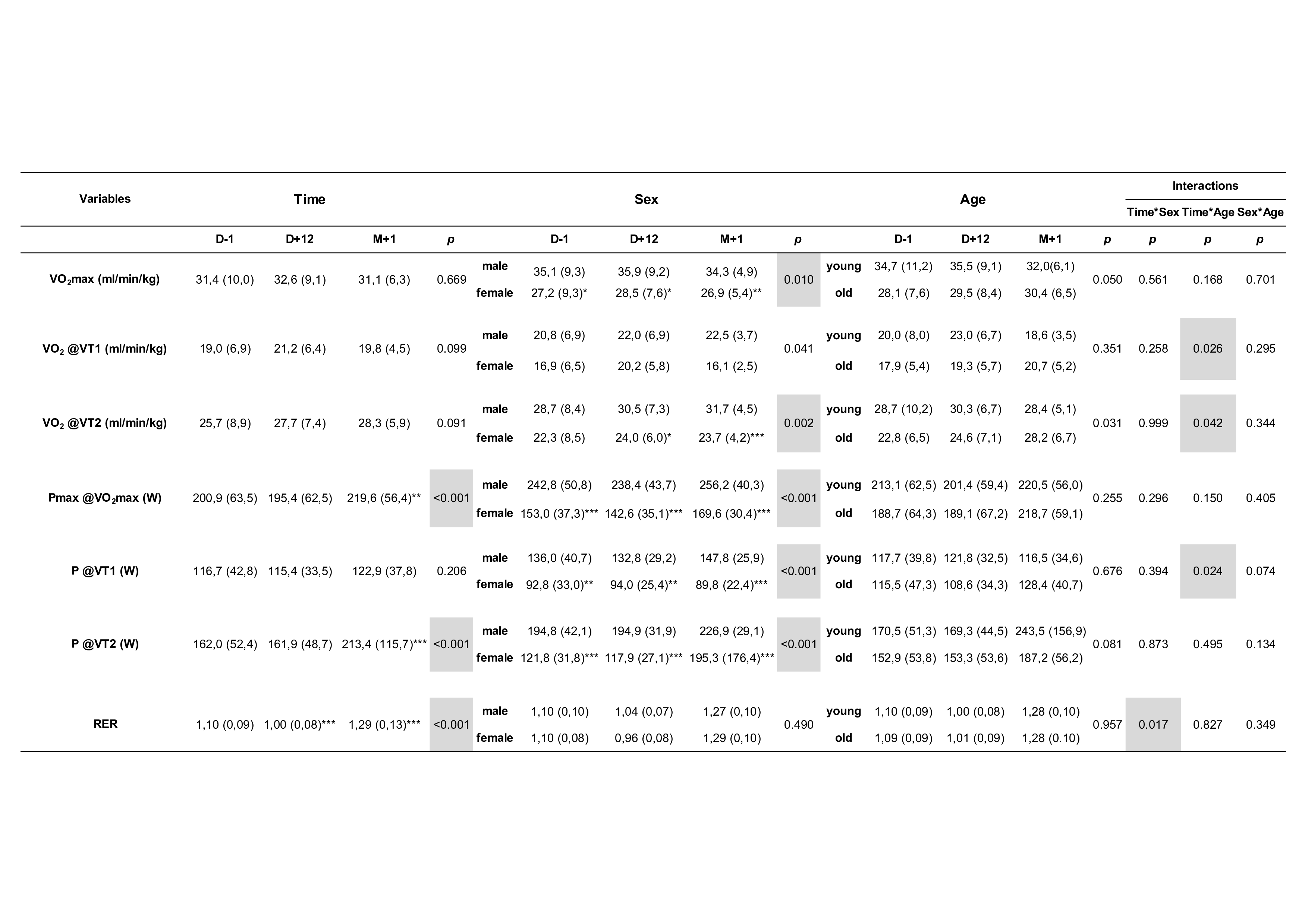

Supplement: Supplementary file 5 — Table S4 Cardiopulmonary exercise testing summary results (mean (SD), overall, and stratified by sex and age). The table presents the effect of time, sex, and age using a repeated‐measures mixed‐effects model. When applicable, pairwise post‐hoc comparisons used a Tukey test to correct for multiple comparisons. For all analyses, significance was accepted at p < 0.05. (Legend for post‐hoc comparisons: D + 12 & D + 30 vs D‐1: * = p < 0.05; ** = p < 0.01; *** = p < 0.001. For Sex and Age variable, post‐hoc comparisons are for each time point). [file JCSM-16-e13773-s003.tiff]

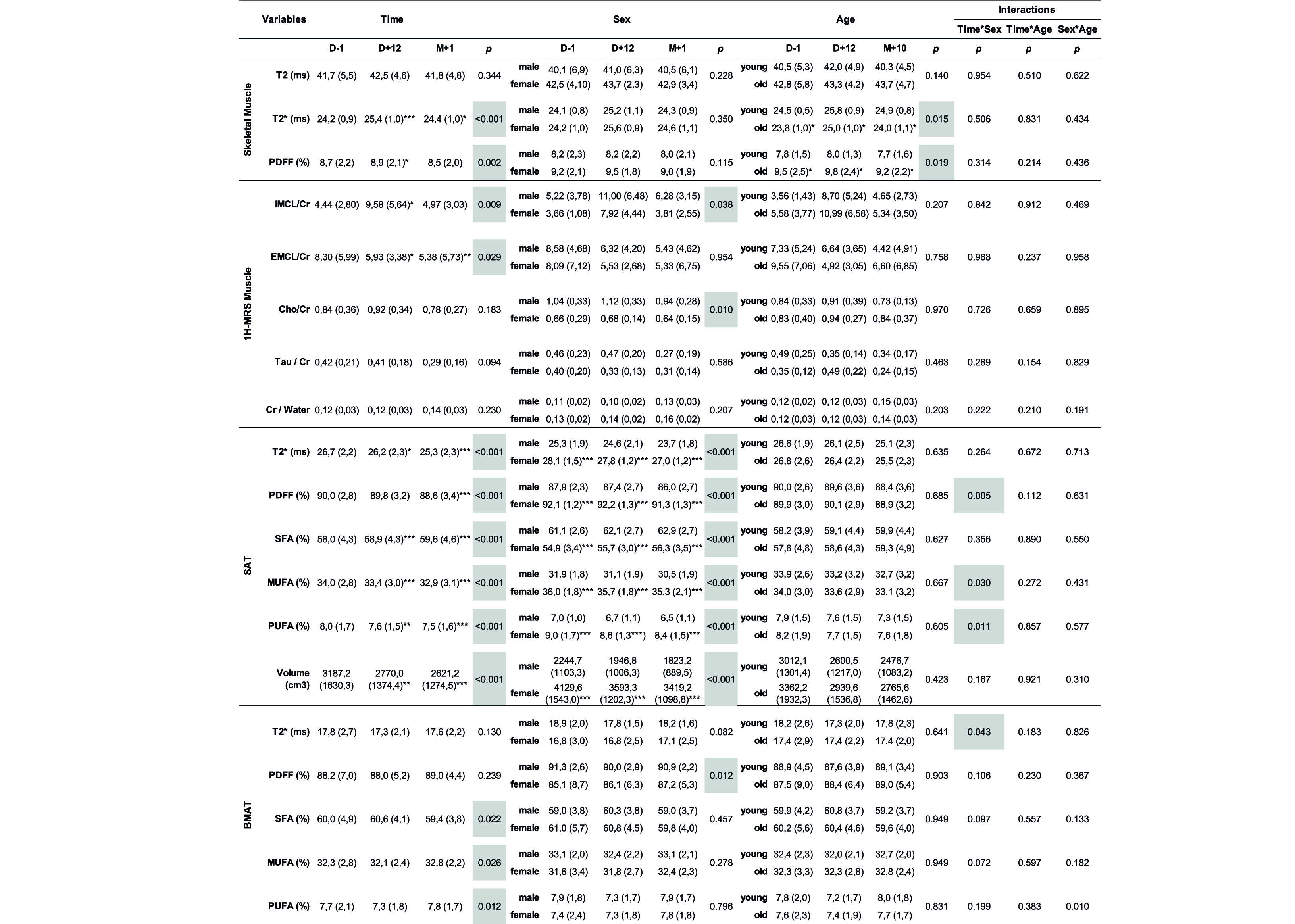

Supplement: Supplementary file 6 — Table S5 Relaxometry and thigh fat composition: 1H‐MRS results (mean (SD), overall, and stratified by sex and age). Analysed tissues are tight muscle, subcutaneous adipose tissue (SAT), and bone marrow adipose tissue (BMAT). The table presents the effect of time, sex, and age, using a repeated‐measures mixed‐effects model. When applicable, pairwise post‐hoc comparisons used a Tukey test to correct for multiple comparisons. For all analyses, significance was accepted at p < 0.05. (Legends for post‐hoc comparisons: comparisons are against D‐1 used as a control for time variable: * = p < 0.05; ** = p < 0.01; *** = p < 0.001. For Sex and Age variable, post‐hoc comparisons are for each time point.) [file JCSM-16-e13773-s009.tiff]

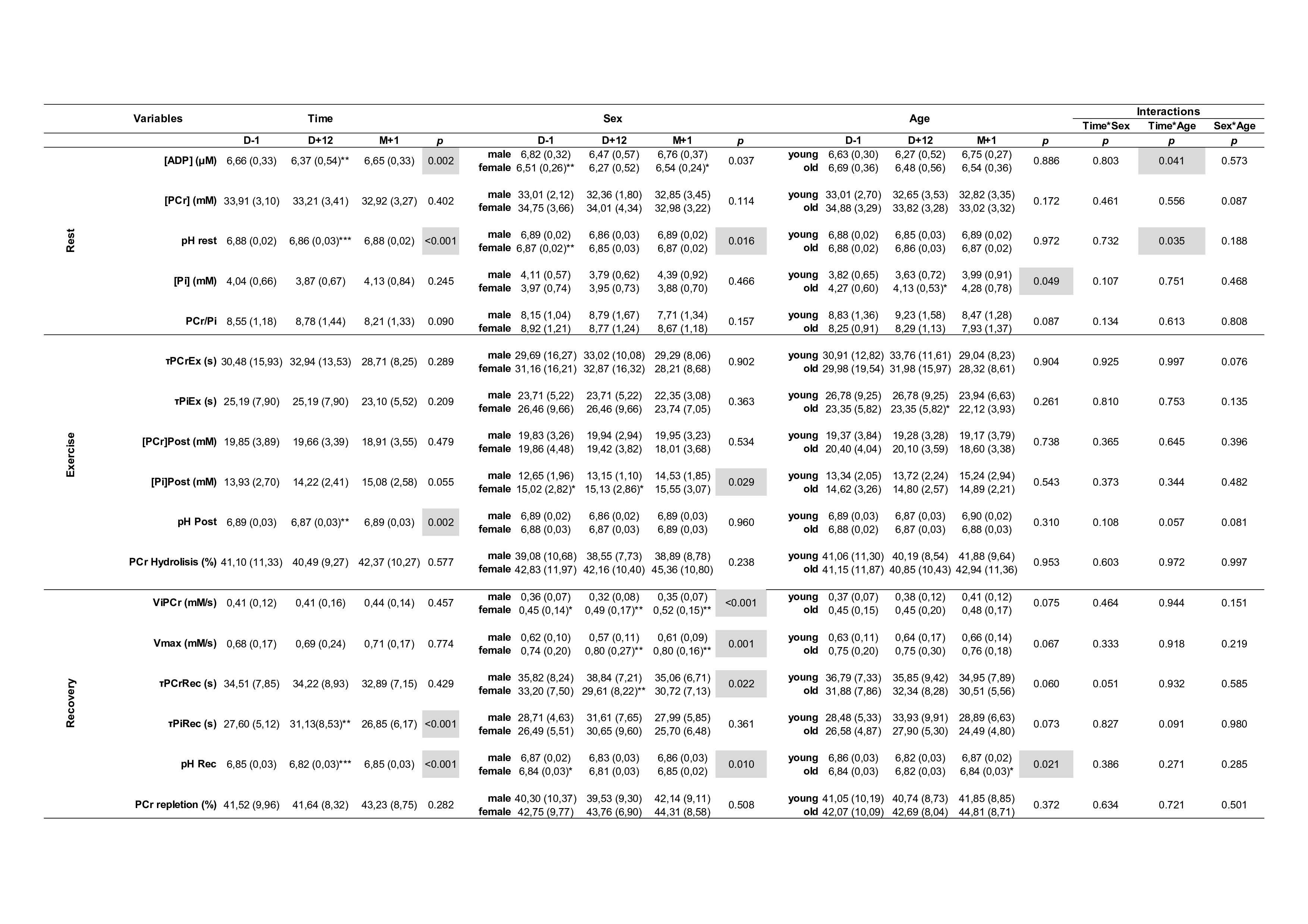

Supplement: Supplementary file 7 — Table S6 31P‐MRS findings at rest, during exercise, and recovery (mean (SD), overall, and stratified by sex and age). The table presents the effect of time, sex, and age, using a repeated‐measures mixed‐effects model. When applicable, pairwise post‐hoc comparisons used a Tukey test to correct for multiple comparisons. For all analyses, significance was accepted at p < 0.05. (Legends for post‐hoc comparisons: for time variable, comparisons are against D‐1 used as a control: * = p < 0.05; ** = p < 0.01; *** = p < 0.001. For Sex and Age variable, post‐hoc comparisons are for each time point.) [file JCSM-16-e13773-s010.tiff]

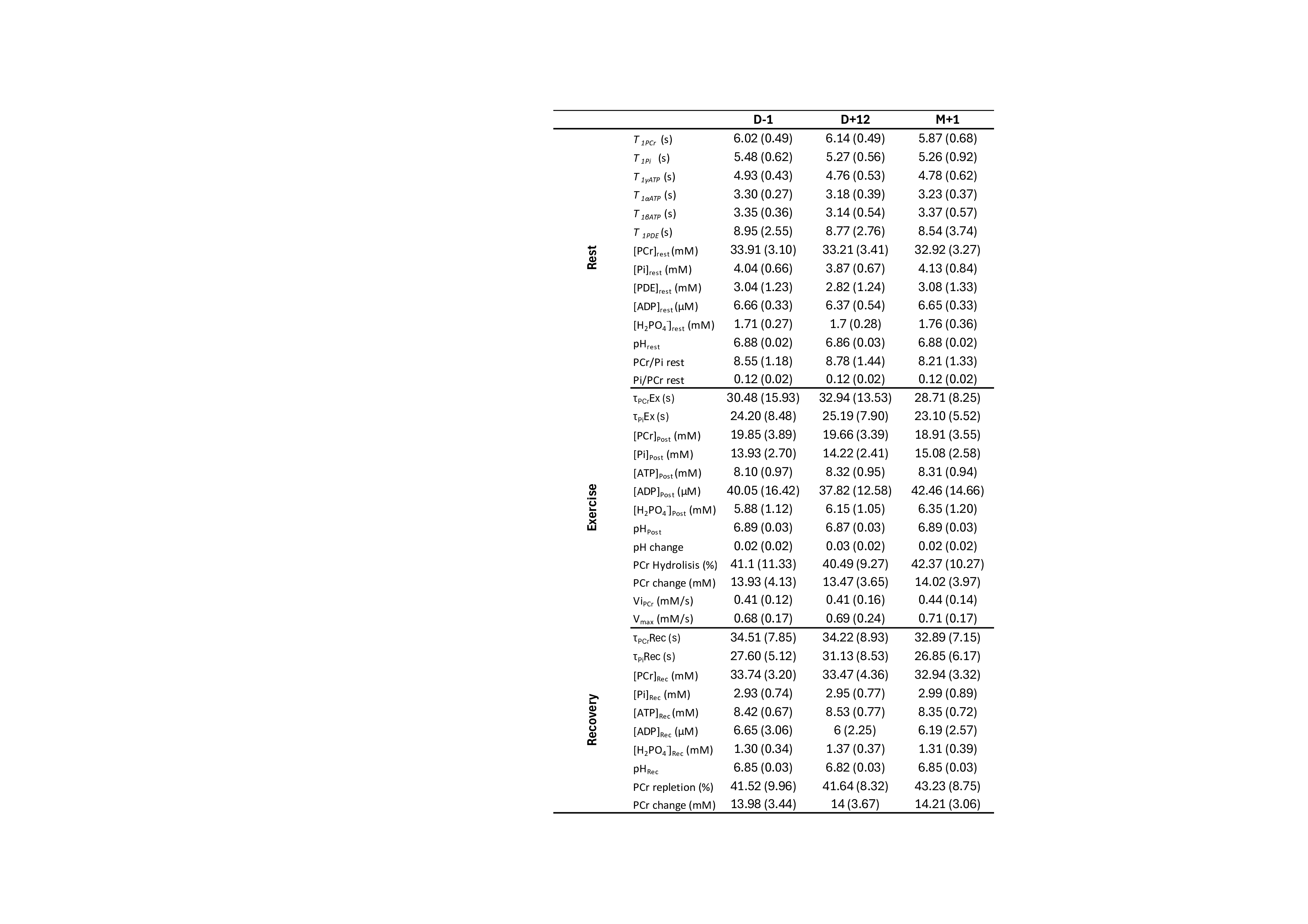

Supplement: Supplementary file 8 — Table S7 Full list of 31P‐MRS quantified biomarkers [file JCSM-16-e13773-s006.tiff]

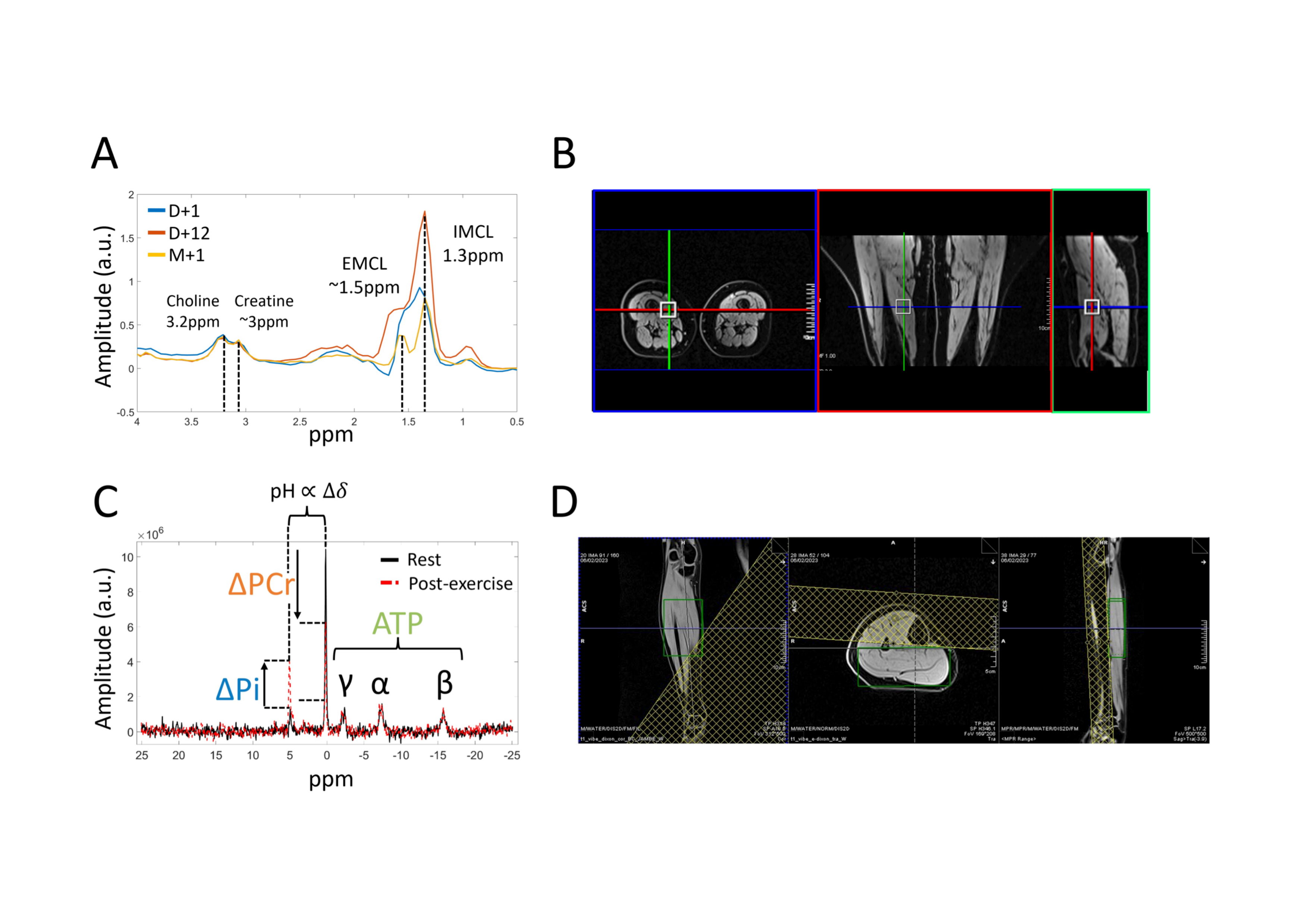

Supplement: Supplementary file 9 — Figure S1 Supporting information [file JCSM-16-e13773-s007.tiff]

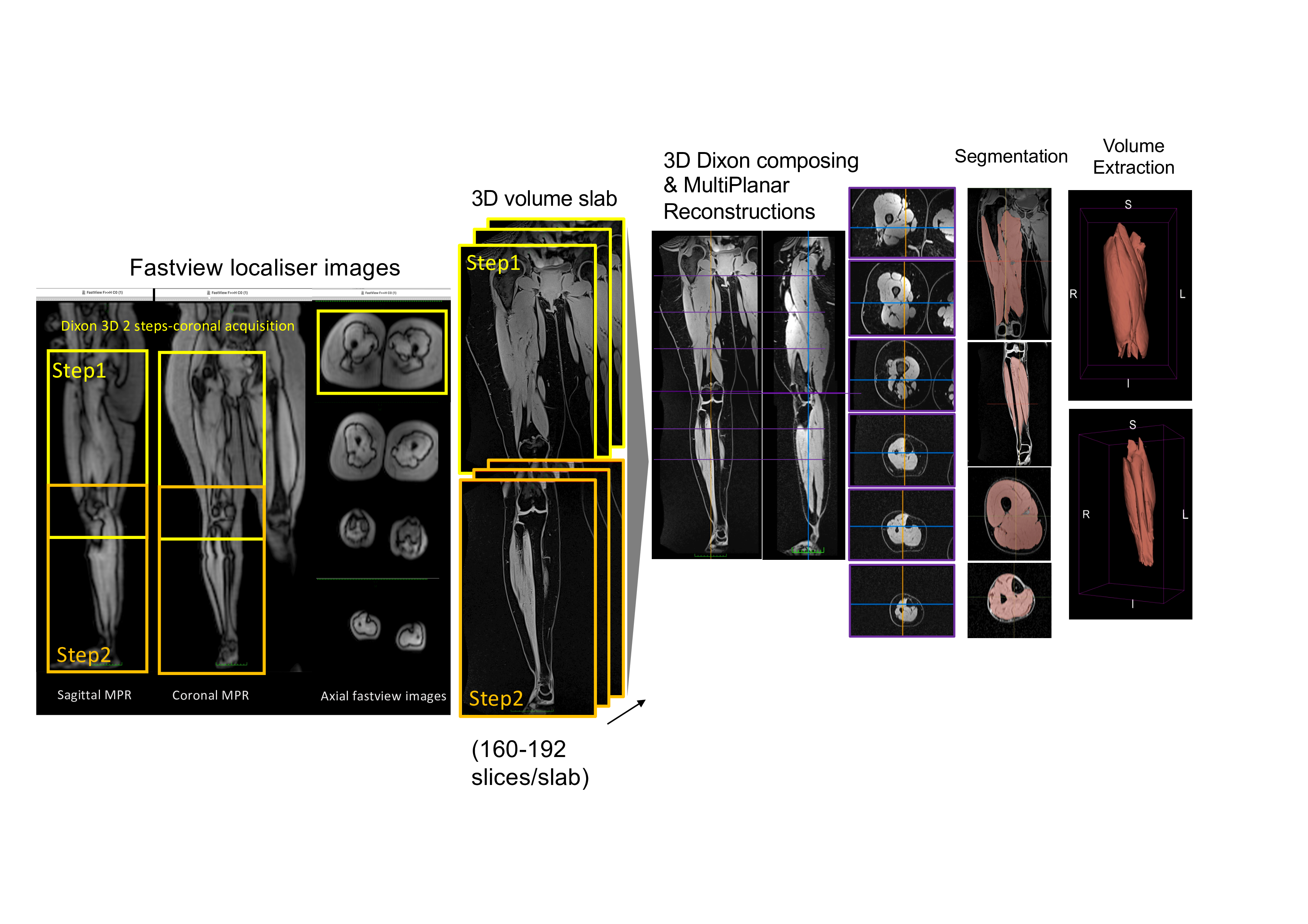

Supplement: Supplementary file 10 — Figure S2A Supporting information [file JCSM-16-e13773-s001.tiff]

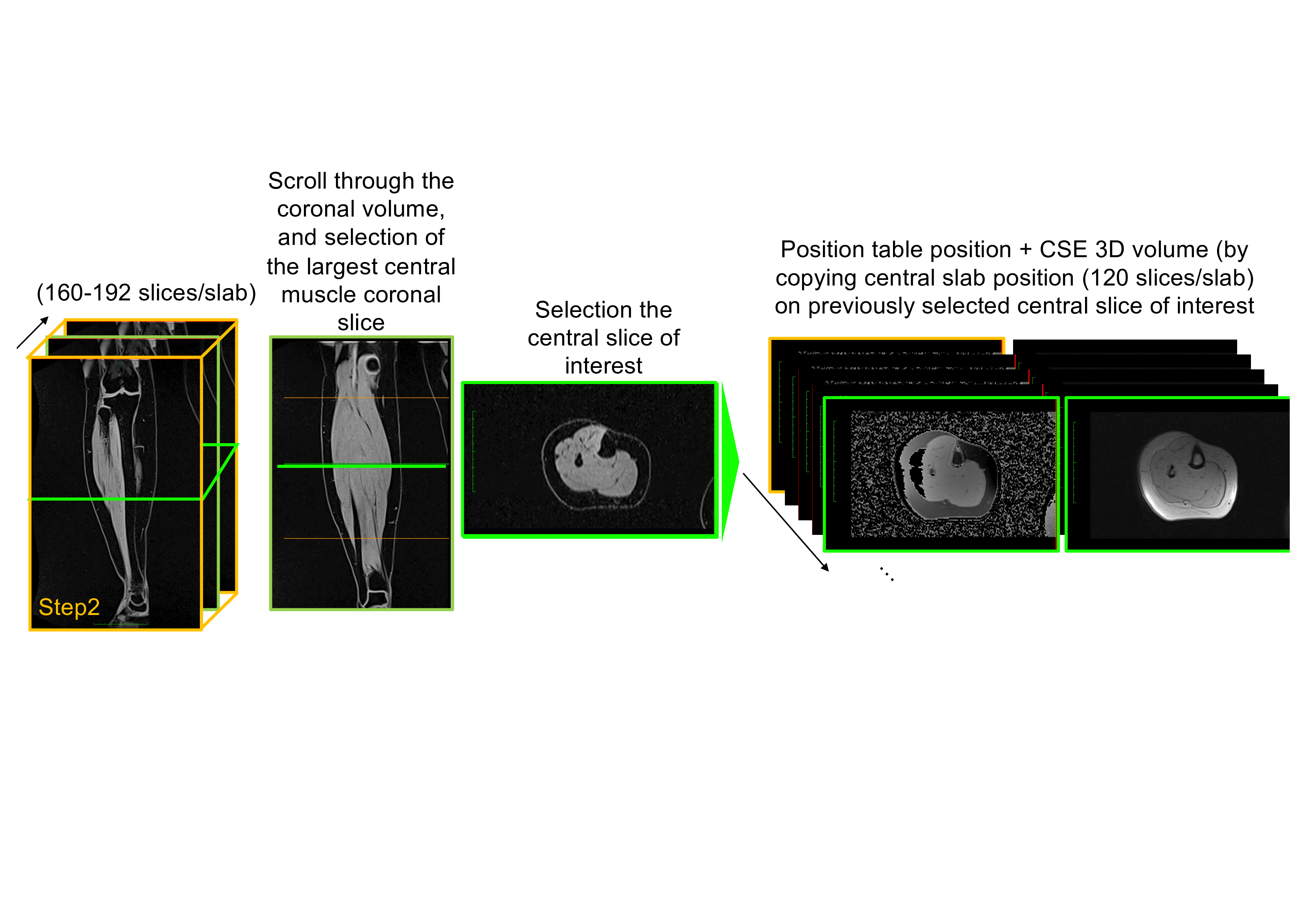

Supplement: Supplementary file 11 — Figure S2B Supporting information [file JCSM-16-e13773-s008.tiff]
